# Supplementary material for: Analyses of 37 composts revealed microbial taxa associated with disease suppressiveness
Source: Appl Environ Microbiol. 2025 Oct 8;91(11):e01100-25. doi: 10.1128/aem.01100-25 (PMC12628690; doi:10.1128/aem.01100-25)
Supplement: Supplemental material — Supplemental methods, Fig. S1 to S17, and Tables S1 to S17. [file aem.01100-25-s0001.pdf]

Supplementary Materials

Additional Material and Methods, Figures and Tables for the paper "*Analyses of 37 composts revealed microbial taxa associated with disease suppressiveness*".

Authors: Anja Logo, Benedikt Boppré, Jacques Fuchs, Monika Maurhofer, Thomas Oberhänsli, Barbara Thürig, Franco Widmer, Johanna Mayerhofer & Pascale Flury

Date: September 22, 2025

Contents

Supplementary Material and Methods **5**

A Physicochemical properties of composts 5

B Microbial activity of composts 5

C Statistical methods for association analysis 6

Supplementary Figures **7**

Supplementary Tables **24**

List of Figures

S1 Growth promotion of composts 1-37 and excluded composts Ex1-3 7

S2 Spearman Rank Sum correlation between relative biomass and disease symptoms rating for the three plant-pathogen-systems 8

S3 Comparison of disease-suppressive activity of the no-compost control in the different experiments 9

S4 Correlation of disease-supressive activity of composts between the plant-pathogens systems 10

S5 Disease-suppressive activity of composts grouped by compost set and composting site 11

S6 Principal Component Analysis based on compost properties 12

S7 Correlation among abiotic and biotic compost properties 13

S8 Correlation plots of mean disease suppression in the cress-*G. ultimum*-system and compost properties 14

S9 Correlation plots of mean disease suppression in the cucumber-*G. ultimum*-system and compost properties 15

S10 Correlation plots of mean disease suppression in the cucumber-*R. solani*-system and compost properties 16

S11 Beta diversity based on Bray-Curtis dissimilarity among all 164 bacterial samples and 162 fungal samples 17

S12 Taxonomic composition of the bacterial communities of the 37 composts 18

S13 Taxonomic composition of the fungal communities of the 37 composts . . 19

S14 Shannon eveness and Shannon diversity index of the bacterial communities and fungal communities 20

S15 Bacterial and fungal community structure and disease-suppressive activity 21

S16 Number of ASVs indicative of disease-suppressive composts identified by different statistical methods in the three plant-pathogen systems 22

S17 Prevalence of indicative ASVs 23

List of Tables

S1 Overview composting process for the different composting sites 24
S2 Compost properties of the 40 composts (1-37, Ex1-3) 25
S3 Number of sequences and ASVs for the different filtering steps of sequencing data 26
S4 Differences in disease-suppressive activity among composts and no-compost controls 27
S5 Five most common ASV classifications at the phylum, family, and genus levels for the 19,129 bacterial and 2,309 fungal ASVs identified across the 37 composts 28
S6 ANOVA tables for differences in alpha diversity metrics among composts. 29
S7 Relationships between compost properties and microbial activity with the bacterial and fungal communities. 30
S8 Relationship between compost sets and composting sites with physicochemical properties, microbial activities, and alpha- and beta-diversity of bacterial and fungal communities 31
S9 Taxonomic classification of indicative ASVs 32
S10 Indicative ASVs which had a sequence match of at least 99% with one of the 75 indicative ASVs in Mayerhofer et al. (2021) 33
S11 Effect of compost age and disease suppression of *G. ultimum* in cress combined on bacterial community structure of the 17 compost published by Mayerhofer et al. (2021) 34
S12 Genera assigned to indicative ASVs for which their presence was previously reported in compost 35
S13 Genera assigned to indicative ASVs for potential involvement in disease suppression was previously reported 37
S14 Fungal ASVs indicative of the nine most suppressive composts 39
S15 Bacterial ASVs indicative of the nine most suppressive composts in the cress-*G. ultimum* system 40
S16 Bacterial ASVs indicative of the nine most suppressive composts in the cucumber-*G. ultimum* system. 44

S17 Bacterial ASVs indicative of the nine most suppressive composts in the cucumber-*R. solani* system. 48

Supplementary Material and Methods

A) Physicochemical properties of composts

Water and $CaCl_2$-extracts were prepared by adding 50 g of fresh compost to 500 mL of deionized water or to 500 mL 0.01 M $CaCl_2$, and shaking horizontally at 75 rpm for 60 min. The pH of the compost was measured in the unfiltered $CaCl_2$-extract directly after shaking using a pH-meter (SevenEasy, Mettler Toledo, Columbus, OH, USA). Both extracts were filtered through cellulose filter paper (MN 619, Machery-Nagel, Düren, Germany). The salinity was assessed by measuring the electrical conductivity in the water-extract (FiveGo Conductivity Meter, Mettler-Toledo, Columbus OH, USA) and converting to compost salinity using the following formula: salinity [$KCl_{equivalent}$ (kg DS)$^{-1}$] = electrical conductivity [mS cm^{-1}] x 583.4 / DS [%]. The content of soluble humic substances (OD_{550}), which can be used as an indicator for compost maturation (Oshins et al., 2022), was measured in the water-extract using a spectrophotometer at a wavelength of 550 nm (Genesys 150, ThermoFisher Scientific, Waltham, MA, USA). Mineral nutrients of the $CaCl_2$-extracts were determined with the Berthelot's reagent for ammonium (Krom, 1980), the cadmium-reduction method (Gal et al., 2004) for nitrate and nitrite and Molybdenum blue method (Drummond and Maher, 1995) for PO_4^{3-}-P and measured using Smartchem 450 Discrete Analyser (AMS Alliance, Guidonia, Italy).

B) Microbial activity of composts

To assess the microbial activity of the composts, the water content of compost samples was adjusted to 60% of the maximum WHC and the samples were incubated for equilibration at 25°C for seven days. FDA hydrolysis [μg FDA (min g DS)$^{-1}$] of composts was assessed by shaking 1 g of compost in 20 mL phosphate buffer (60 mM, pH 7.6) containing 400 mg FDA (98%, Aldrich, USA) at 90 rpm for 20 min. The reaction was stopped by adding 20 mL Acetone (99.5%, Roth, Germany). Fluorescence was measured with a fluorescence spectrometer (excitation at 490 nm, emission at 525 nm, TECAN infinite 200 PRO, Switzerland). A negative control sample without the addition of FDA was included in the analysis.

To assess basal respiration of the composts, 10 g dry substance equivalent of fresh compost was weighed into perforated 25 mL centrifuge tubes and placed in 250 mL Schott bottles in the presence of 0.1 M NaOH as a CO_2-trap. After a 24 h incubation period at 25°C the NaOH in the bottle was replaced with 40 mL of fresh NaOH and further incubated for 120 h with once exchanging the NaOH after 48 h. The remaining NaOH was determined by automated titration (808 Titrando, Methorm, Zofingen, Switzerland) with 0.1M HCl after precipitation of absorbed $C0_2$ by excess $BaCl_2$. Based on the amount of acid used for titration, the respiration [mg CO^2-C $(h)^{-1}$] was calculated according to Alef and Nannipieri (1995) and averaged over the two incubation periods.

C) Statistical methods for association analysis

Point Biserial corrleation (setting "r.g") and Indicator Species Analysis ("indVal.g") were applied using the "*multipatt*" function (*Indicspecies*, Cáceres and Legendre (2009)). Differential abundance was assessed using MaAsLin2 and ALDEx2, both of which were recommended in the method comparison study by Nearing et al. (2022). MaAsLin2 was run with default settings except for arcsine square-root transformation and no data scaling (Mallick et al., 2021). For ALDEx2 we employed the "*aldex.clr*" function with log-ratio transformation, followed by "*aldex.test*" for group differences and "*aldex.effect*" for effect sizes (Fernandes et al., 2014). Rarefied read counts were used for PBC, ISA, and MaAsLin2 and unrarefied read counts for ALDEx2.

Supplementary Figures

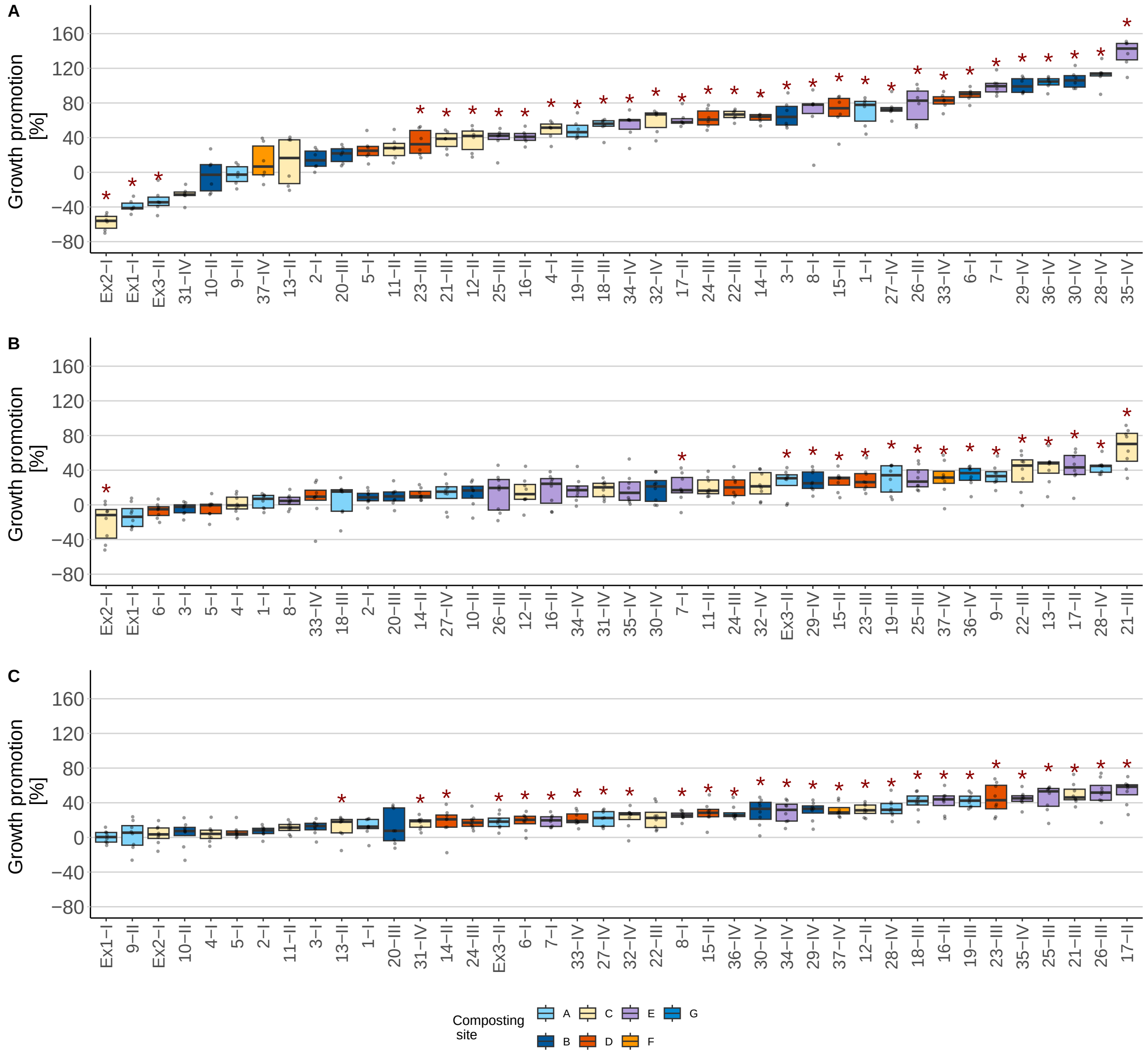

Figure S1: **Growth promotion of composts 1-37 and excluded composts Ex1-3.**: (A) Cress-*G. ultimum*, (B) cucumber-*G. ultimum*, and (C) cucumber-*R. solani*. Growth promotion was calculated by dividing the shoot biomass without pathogen by the average shoot biomass without pathogen of the no-compost control (NC) of the respective compost set. The roman numbers (I-IV) after the compost number indicate the compost set (I = May 2022, II = July 2022, III = September 2022, IV = May 2023). Grey points represent growth promotion for individual pots, boxes show the interquartile range, and dashes denote the median of six replicates for cress or eight replicates for the cucumber systems. Treatments are ordered by increasing mean. Red asterisks indicate significant differences between compost treatments and the no-compost control of the respective set (Tukey HSD test, $p < 0.05$, or pairwise Wilcoxon test, $p < 0.05$). Box colors highlight the composting site.

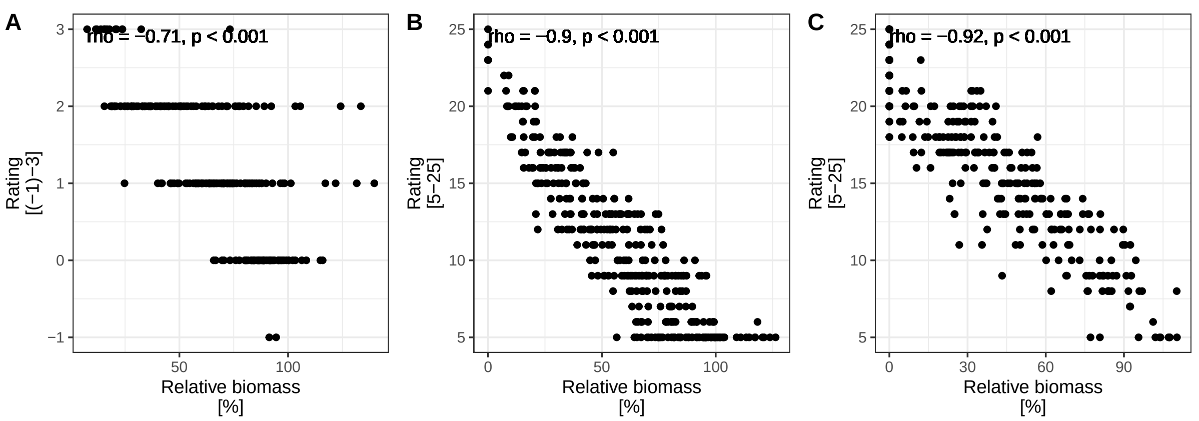

Figure S2: **Spearman Rank Sum correlation between relative biomass and disease symptoms rating for the three plant-pathogen-systems** (A) cress-*G. ultimum*-system (B) cucumber-*G. ultimum*-system and (C) cucumber-*R. solani*-system For pathogen concentrations see caption Figure S3. For the cucumber assays, each seedling was rated separately and was summed for all five seedlings. The following disease ratings were used: cress-*G. ultimum*: -1 better growth than peat without pathogen, 0 healthy, 1 some yellow leaves, reduced growth, 2 half of leaves yellow, growth substantially reduced, 3 majority of leaves yellow, growth extremely reduced; cucumber-*G. ultimum*: 1 healthy, 2 reduced growth, 3 reduced growth, yellow, deformed, 4 death after emergence, 5 not emerged; cucumber-*R. solani*: 1 healthy, 2 brown lesions at the stem but still standing, 3 brown lesions and bend over, 4 dead after emergence, 5 not emerged.

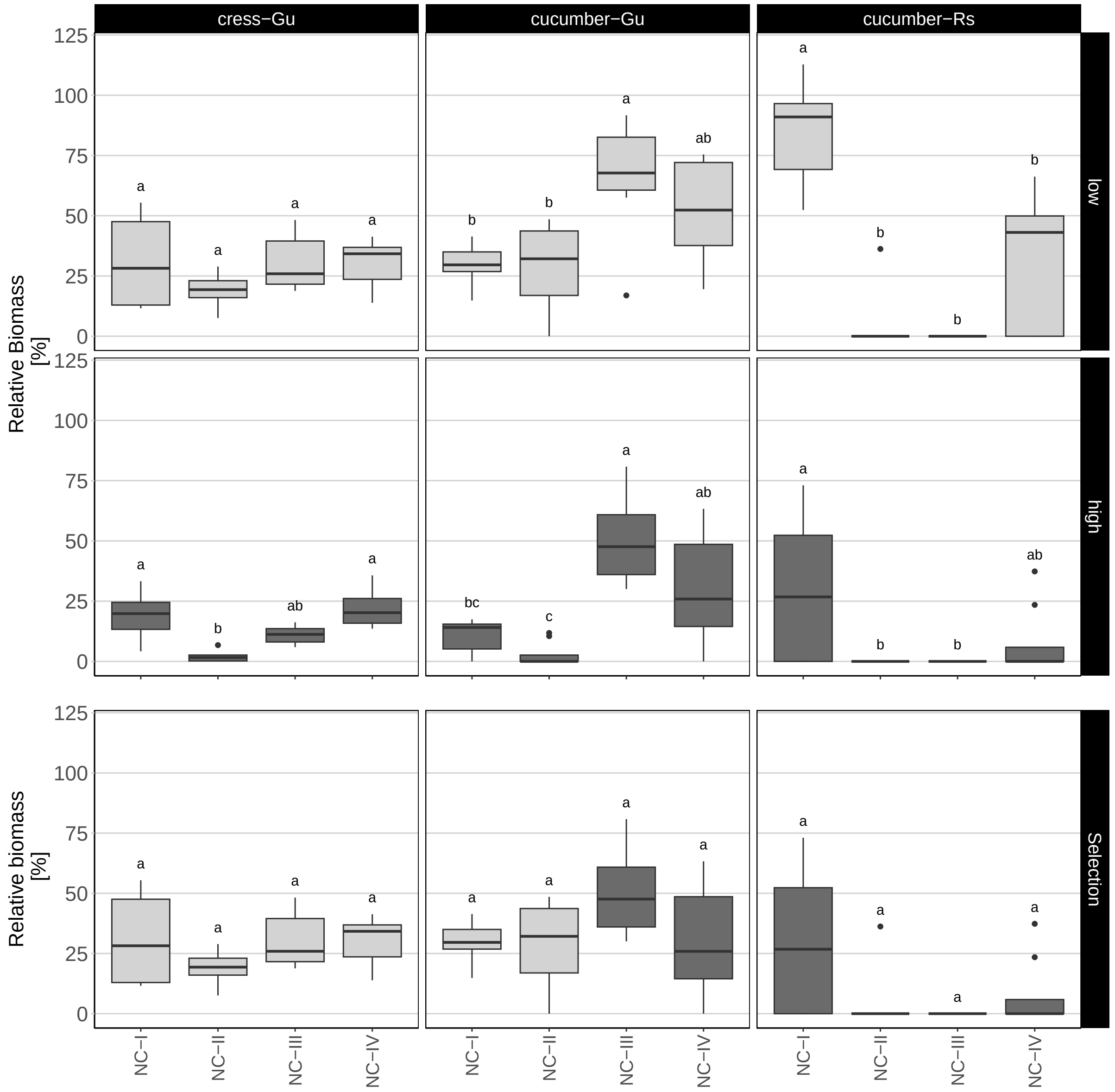

Figure S3: **Comparison of disease-suppressive activity of the no-compost control across experiments**. Pathogen pressure can vary between experiments. Therefore, two pathogen concentrations were tested for all experiments. For *G. ultimum* "low" corresponds to 0.45 g pathogen per liter of substrate and "high" for 1.35 g per liter. For *R. solani* "low" corresponds to three agar plugs with a diameter of 0.8 cm covered with *R. solani* and "high" for 1.4 cm plugs. In batch IV for *R. solani*, "low" corresponds to 0.6 cm plugs, and "high to 0.8 cm plugs. For each compost set, the pathogen concentration was selected to ensure that the relative biomass in the no-compost control was not significantly different among compost sets (ANOVA followed by Tukey HSD test, p <0.05, or Kruskal-Wallis followed by pairwise Wilcoxon test, p <0.05). This resulted in the following concentrations: Cress-*G. ultimum*: 0.45 g *G. ultimum* per liter of substrate in sets I-IV, Cucumber-*G. ultimum*: 0.45 g *G. ultimum* per liter of substrate in sets I and II, and 1.35 g *G. ultimum* per liter of substrate in sets III and IV, cucumber-*R. solani*: three plugs with a diameter of 0.8 cm in sets II–IV and 1.4 cm in set I.

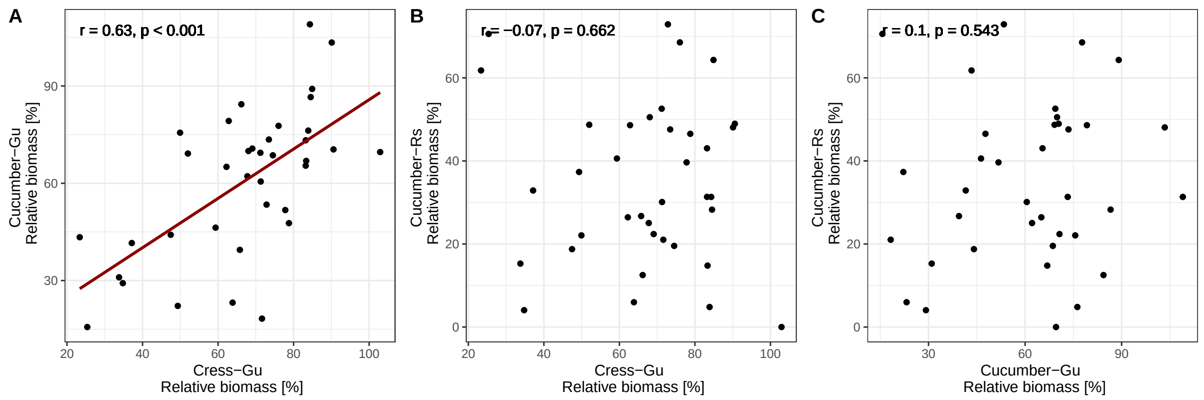

Figure S4: **Correlation of disease-supressive activity of composts between the plant-pathogens systems:** (A) cress-*G. ultimum* and cucumber-*G. ultimum*, (B) cress-*G. ultimum* and cucumber-*R. solani*, (C) cucumber-*G. ultimum* and cucumber-*R. solani*. Disease suppression is shown as relative biomass calculated by dividing the shoot biomass per pot with pathogen by the average shoot biomass of all pots of the same treatment without pathogen.

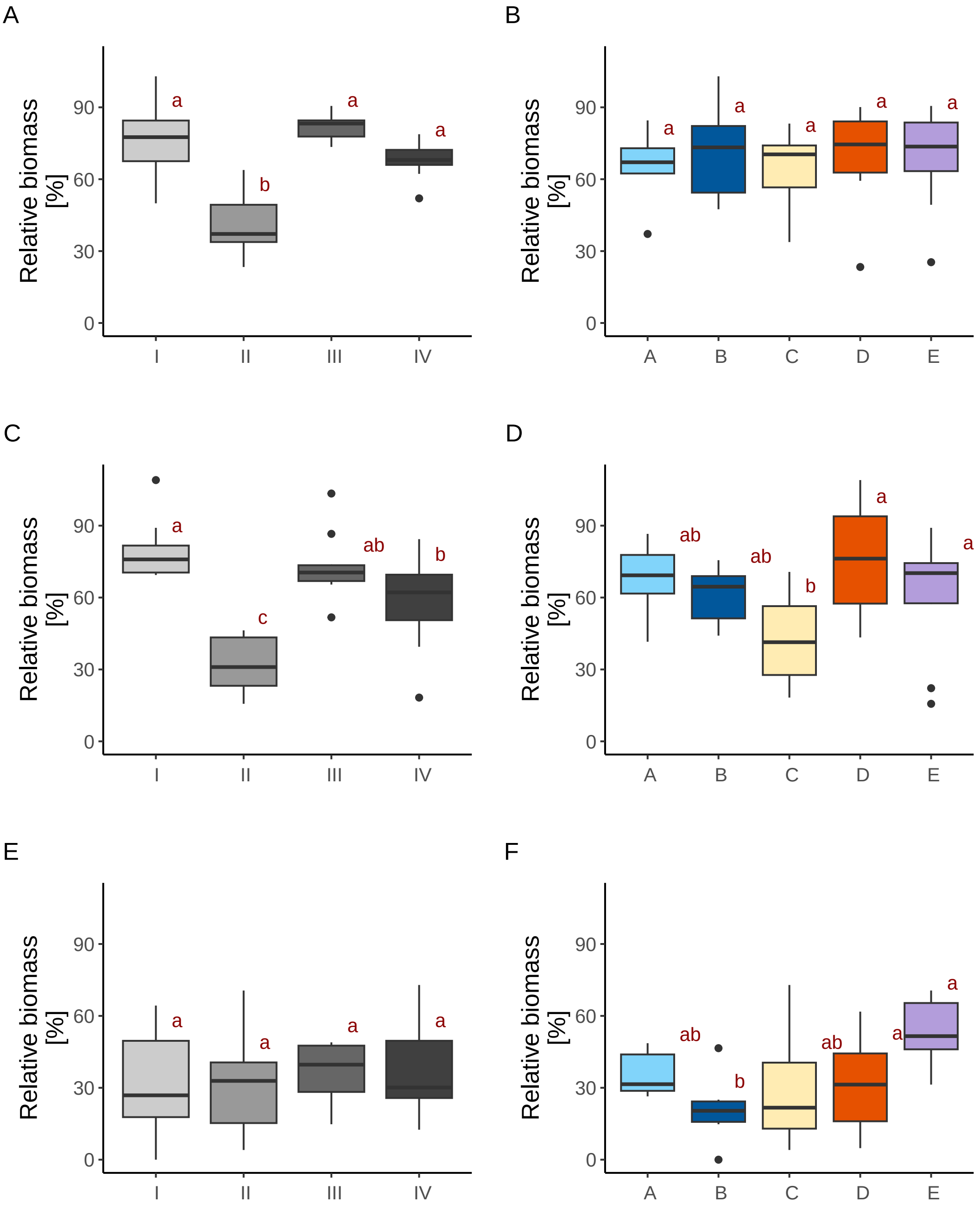

Figure S5: **Disease-suppressive activity of composts grouped by (A, C, E) compost set and (B, D, F) composting site** for (A, B) cress-*G. ultimum*, (C, D) cucumber-*G. ultimum* and (E, F) cucumber-*R. solani*. Composts originating from sites F and G had only one sample each. Therefore, these two composts (36, 37) were excluded from the analysis. Boxes show the interquartile range and dashes denote the median. Groups sharing the same letter within a plot are not statistically different (ANOVA followed by pairwise Tukey HSD test (p <0.05)). Statistics: compost set from top to bottom: F = 21.97, p <0.001, F = 16.79, p <0.001, F = 0.34, p = 0.799 and composting site: F = 0.18, p = 0.948, F = 2.43, p = 0.07, F = 3.43, p = 0.02

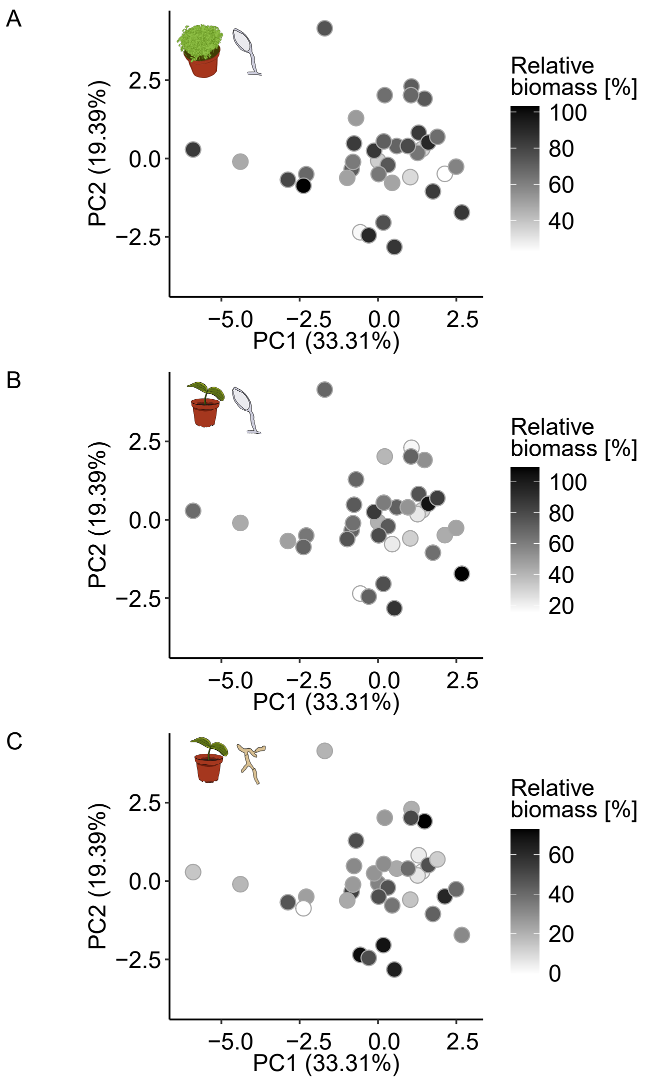

Figure S6: **Principal Component Analysis based on compost properties.** The Euclidean distance between composts was calculated based on nine selected compost properties (indicated with an asterisk in Table 1). Strongly co-correlated compost properties ($|\rho|$ >0.8, Supplementary Figure S7) and properties with missing data (basal respiration, compost age) or which were calculated from other properties (nitrification index, N_{min}, C_{org}/N_{tot}) were removed. The plots display the first and second principal component. Dots represent composts colored according to their disease suppression in (A) the cress-*G. ultimum*-system, (B) the cucumber-*G. ultimum*-system and (C) the cucumber-*R. solani*-system.

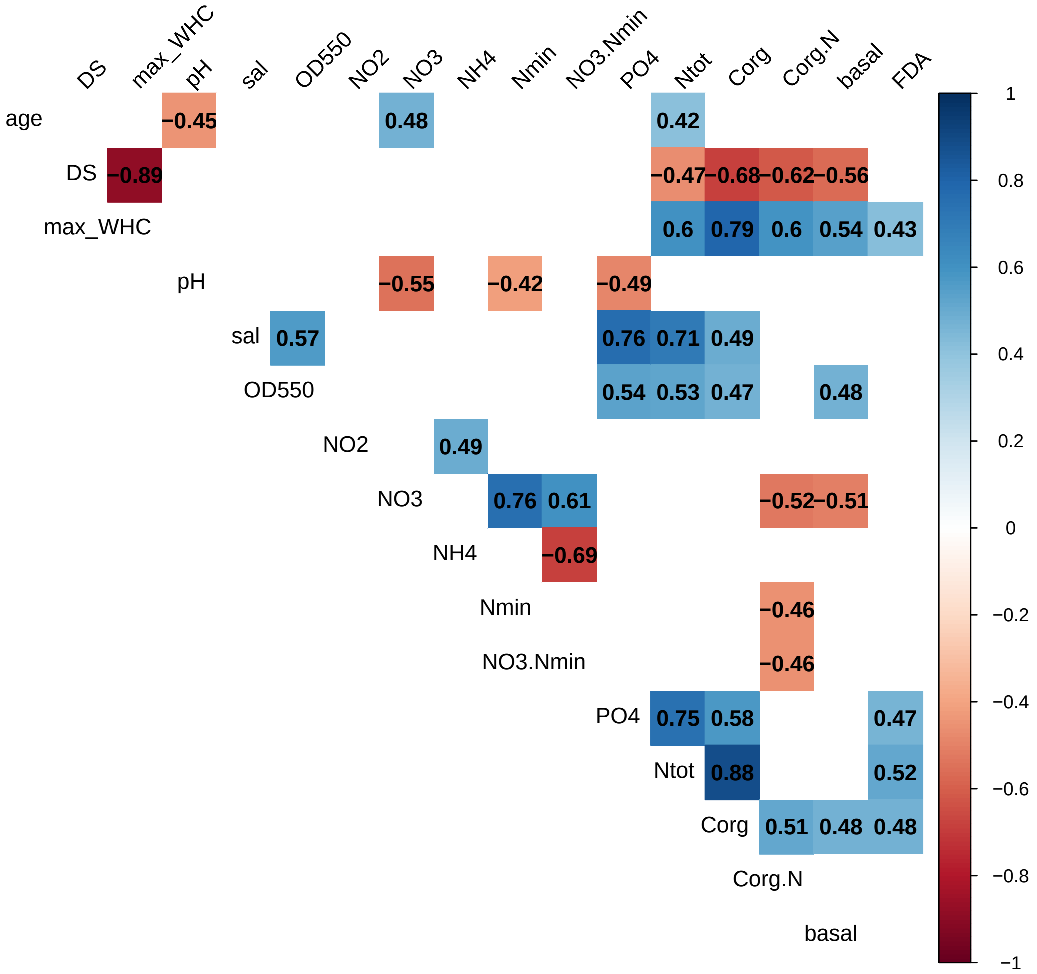

Figure S7: **Correlation among abiotic and biotic compost properties**. *P*-values were adjusted for multiple testing using Benjamini & Hochberg correction. Pearson's correlation was used when both properties were normally distributed, otherwise Spearman's Rank sum test.

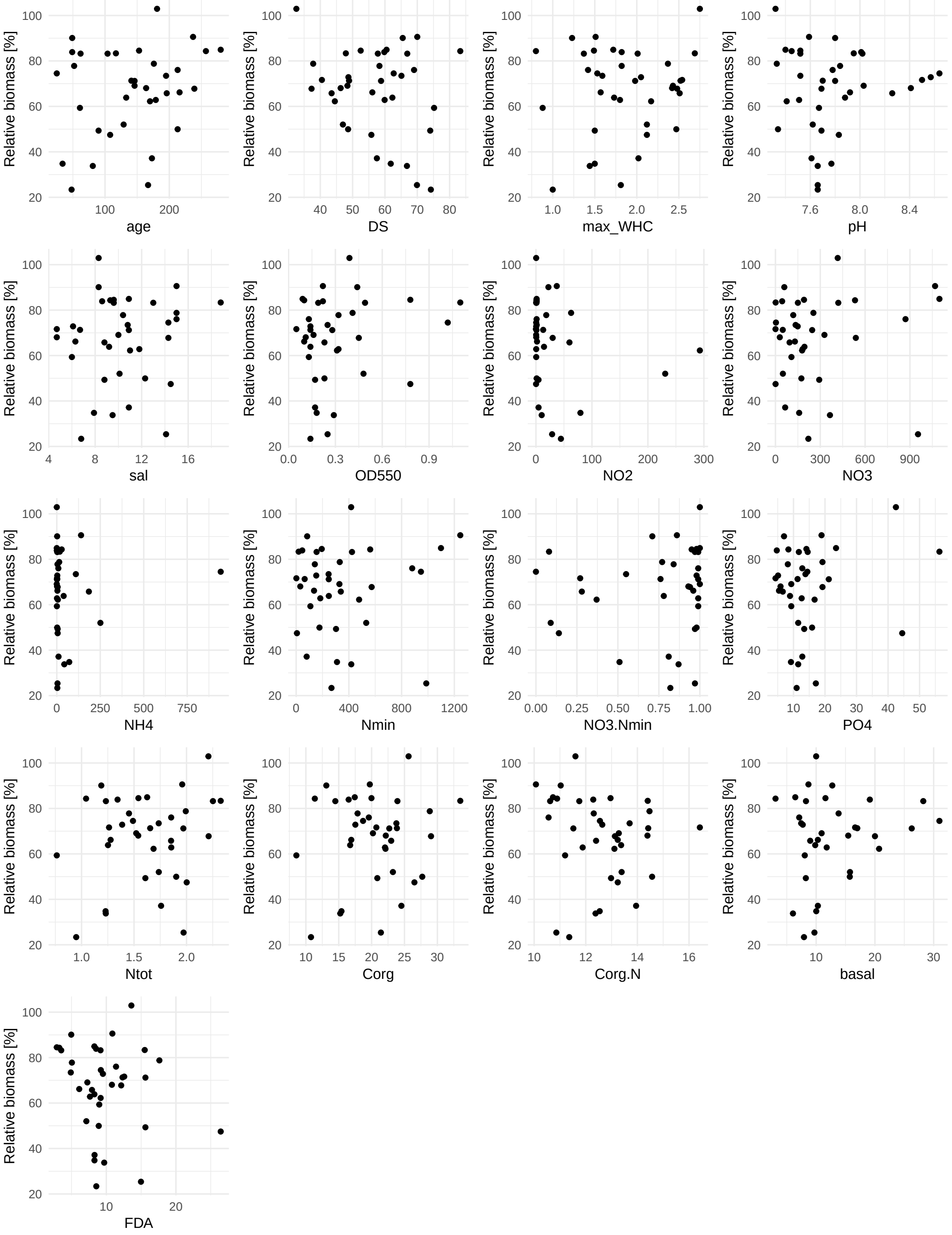

Figure S8: **Correlation plots of mean disease suppression in the cress-*G. ultimum*-system and compost properties** such as compost age [days], dry substance (DS) [%], water holding capacity [g H_20 (g DS)$^{-1}$], pH, salinity (sal) [g KCl_{eq} (kg DS)$^{-1}$], soluble humic substances (OD_{550}), NO_2^--N [mg (kg DS)$^{-1}$] ,NO_3^--N [mg (kg DS)$^{-1}$], NH_4^+-N [mg (kg DS)$^{-1}$], total mineral nitrogen (N_{min}) [mg (kg DS)$^{-1}$], NO_3^--N/N_{min}-ratio, PO_4^{3-}-P [mg (kg DS)$^{-1}$], total nitrogen (N_{tot}) [%], total organic carbon (C_{org}) [%], C_{org}/N_{tot}, basal respiration (basal) [mg CO^2-C (h)$^{-1}$], FDA hydrolysis [μg FDA (min g DS)$^{-1}$]. Correlations were based on 37 composts, except for compost age 35 and basal respiration only 34 were available. Statistics are shown in Table 2.

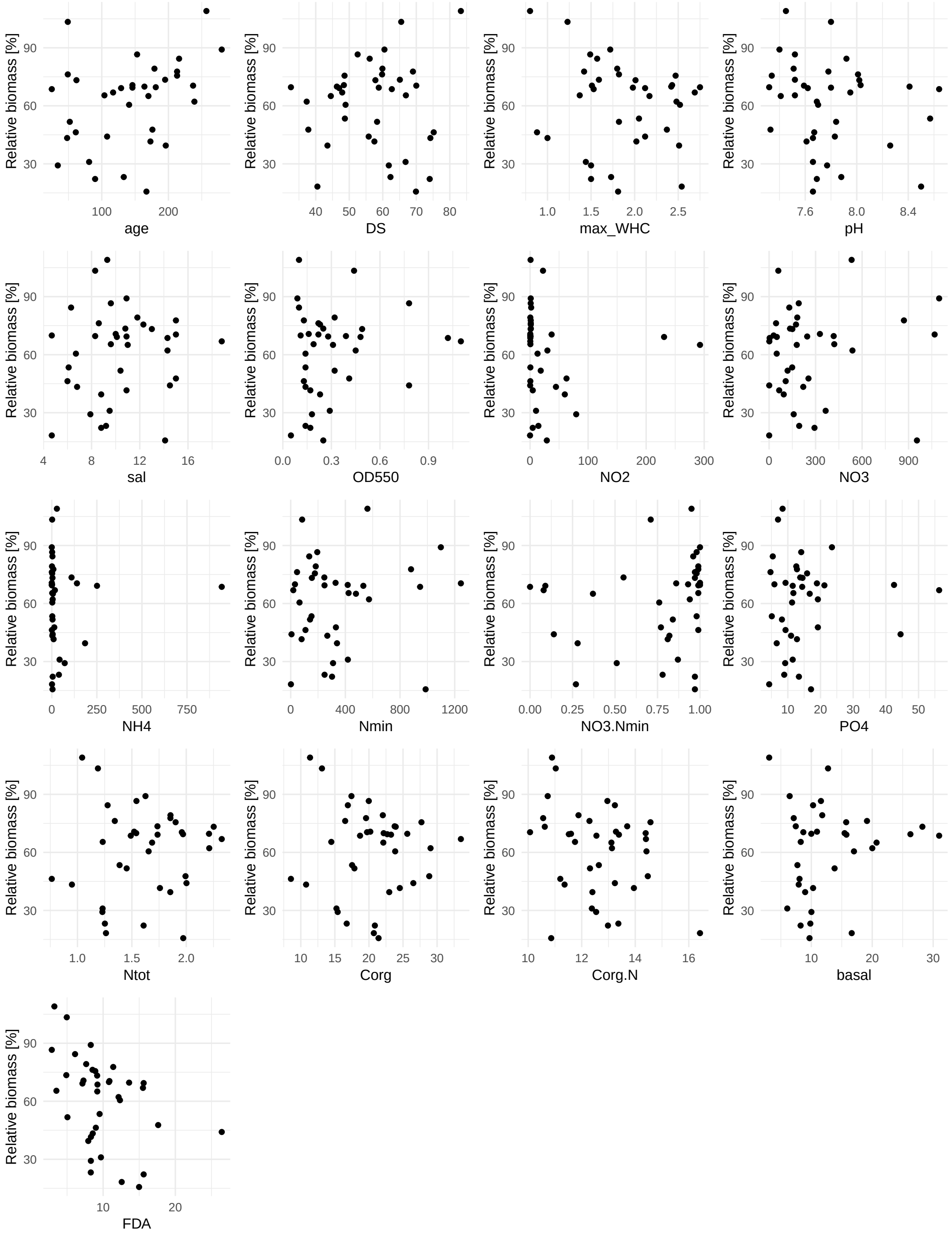

Figure S9: **Correlation plots of mean disease suppression in the cucumber-*G. ultimum*-system and compost properties** such as compost age [days], dry substance (DS) [%], water holding capacity [g H_20 (g DS)$^{-1}$], pH, salinity (sal) [g KCl_{eq} (kg DS)$^{-1}$], soluble humic substances (OD_{550}), NO_2^--N [mg (kg DS)$^{-1}$] ,NO_3^--N [mg (kg DS)$^{-1}$], NH_4^+-N [mg (kg DS)$^{-1}$], total mineral nitrogen (N_{min}) [mg (kg DS)$^{-1}$], NO_3^--N/N_{min}-ratio, PO_4^{3-}-P [mg (kg DS)$^{-1}$], total nitrogen (N_{tot}) [%], total organic carbon (C_{org}) [%], C_{org}/N_{tot}, basal respiration (basal) [mg CO^2-C (h)$^{-1}$], FDA hydrolysis [μg FDA (min g DS)$^{-1}$]. Correlations were based on 37 composts, except for compost age 35 and basal respiration only 34 were available. Statistics are shown in Table 2.

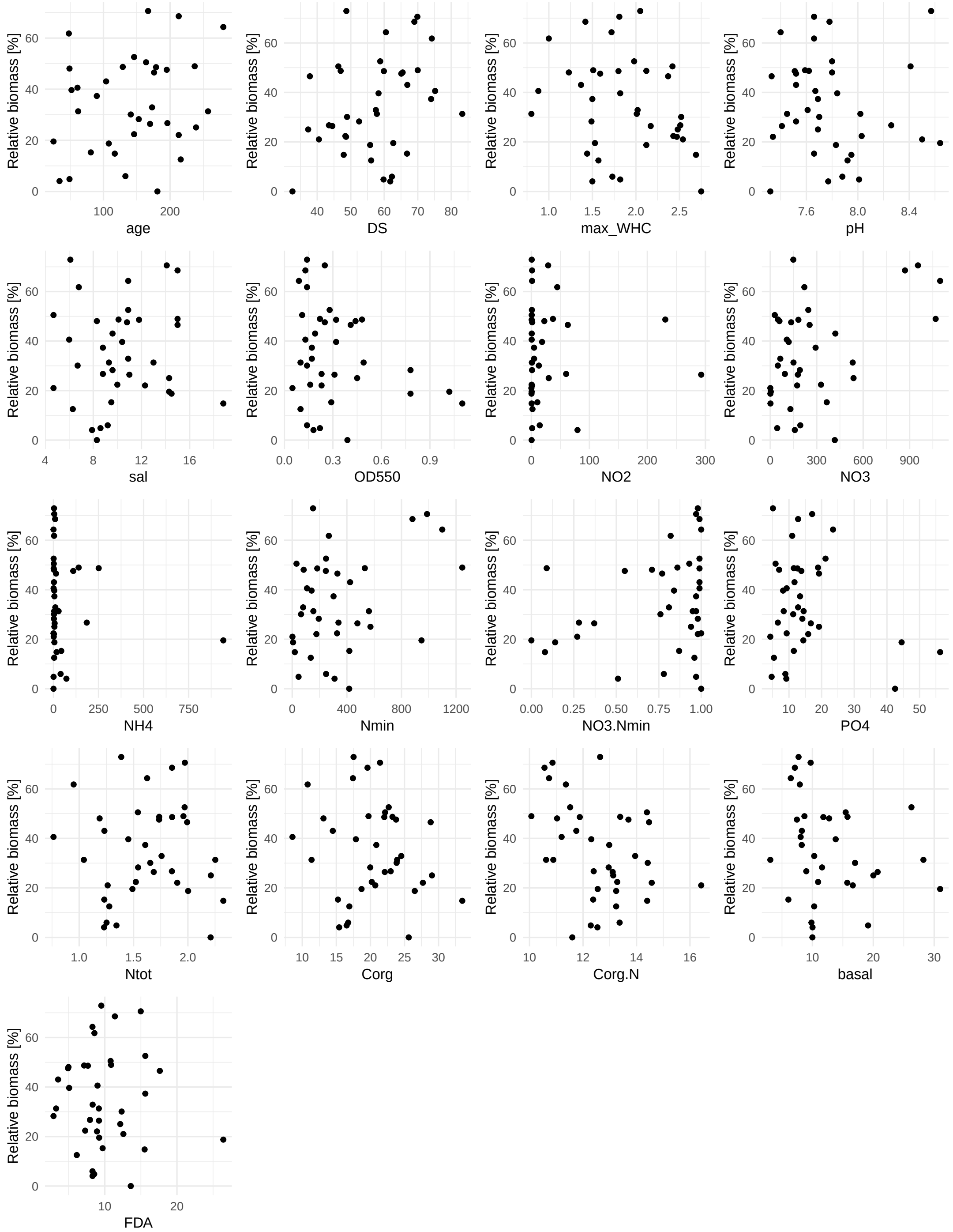

Figure S10: **Correlation plots of mean disease suppression in the cucumber-*R. solani*-system and compost properties** such as compost age [days], dry substance (DS) [%], water holding capacity [g H_20 (g DS)$^{-1}$], pH, salinity (sal) [g KCl_{eq} (kg DS)$^{-1}$], soluble humic substances (OD_{550}), NO_2^--N [mg (kg DS)$^{-1}$] ,NO_3^--N [mg (kg DS)$^{-1}$], NH_4^+-N [mg (kg DS)$^{-1}$], total mineral nitrogen (N_{min}) [mg (kg DS)$^{-1}$], NO_3^--N/N_{min}-ratio, PO_4^{3-}-P [mg (kg DS)$^{-1}$], total nitrogen (N_{tot}) [%], total organic carbon (C_{org}) [%], C_{org}/N_{tot}, basal respiration (basal) [mg CO^2-C (h)$^{-1}$], FDA hydrolysis [μg FDA (min g DS)$^{-1}$]. Correlations were based on 37 composts, except for compost age 35 and basal respiration only 34 were available. Statistics are shown in Table 2.

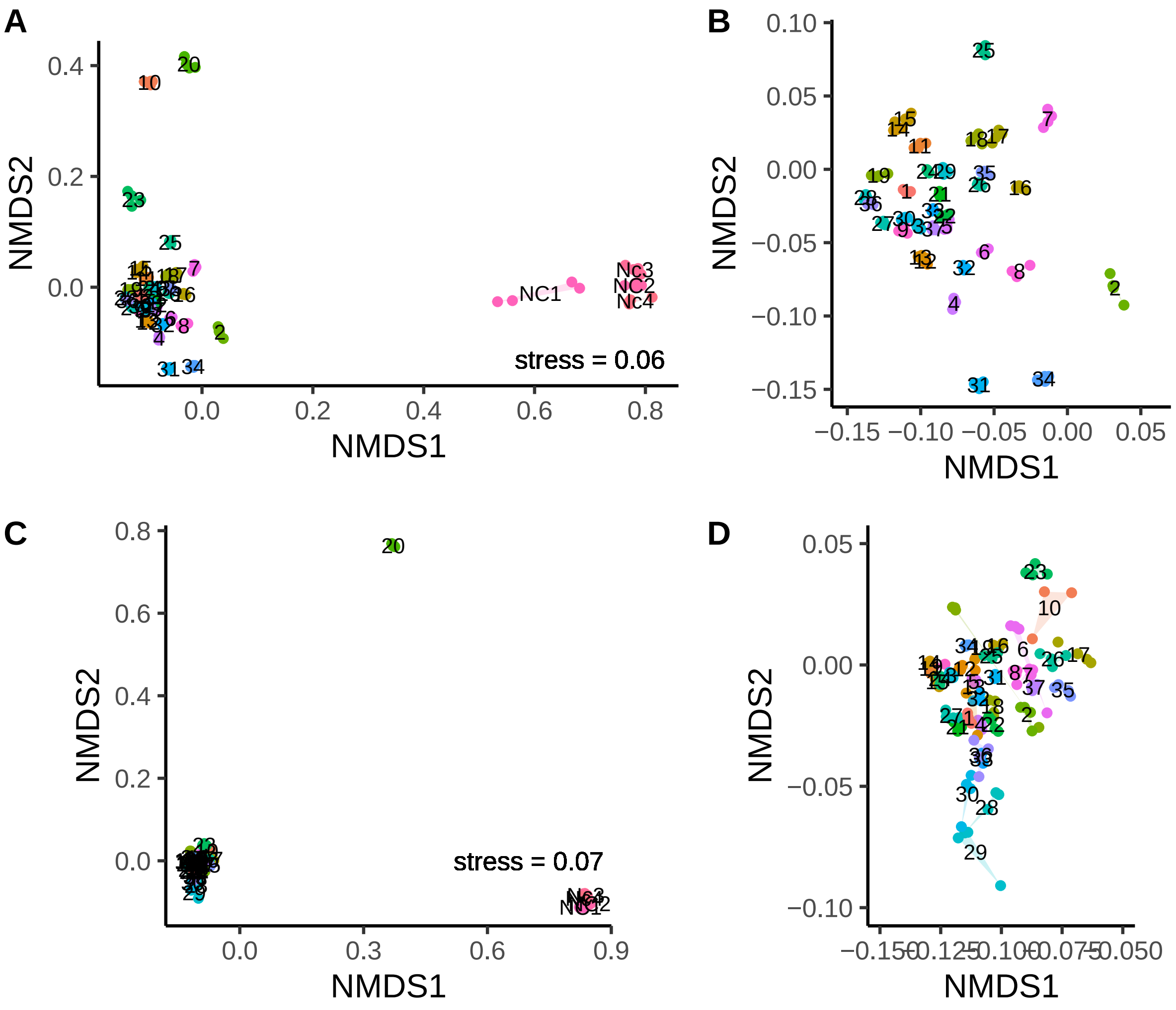

Figure S11: **Beta diversity based on Bray-Curtis dissimilarity was assessed among all (A, C) 164 bacterial samples and (C, D) 162 fungal samples**. The samples are colored by substrate ID (compost 1-17, non-compost control NC 1-4). The four replicates of each substrate are connected to form a polygon. The left plot displays all samples, while the right plot provides a zoomed-in view of the point cloud containing the majority of the samples.

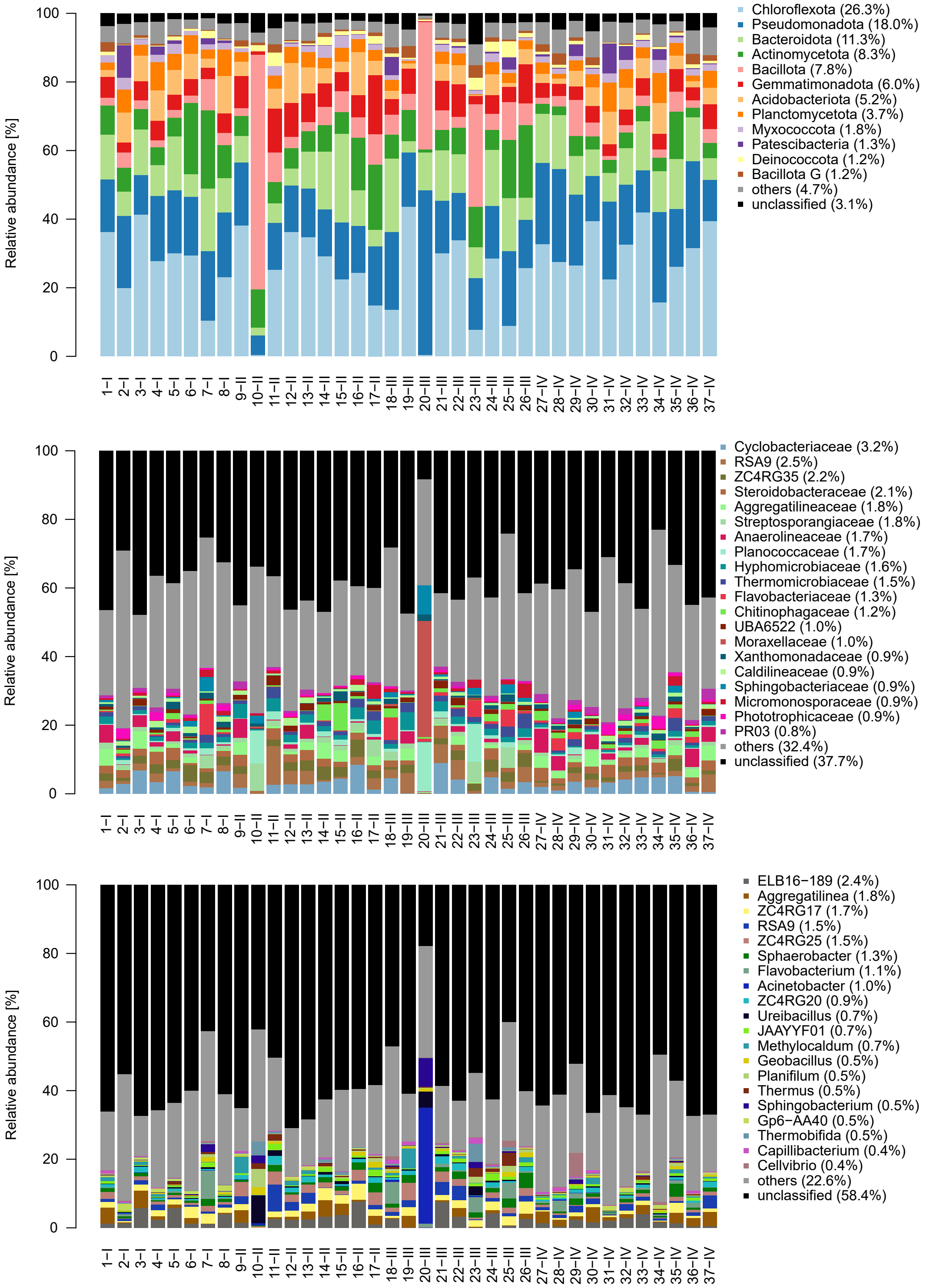

Figure S12: **Taxonomic composition of the bacterial communities of the 37 composts** at (A) phylum, (B) family and (C) genus level. Taxonomic composition was calculated based on rarefied read numbers and averaged for the sequencing replicates (n=4).

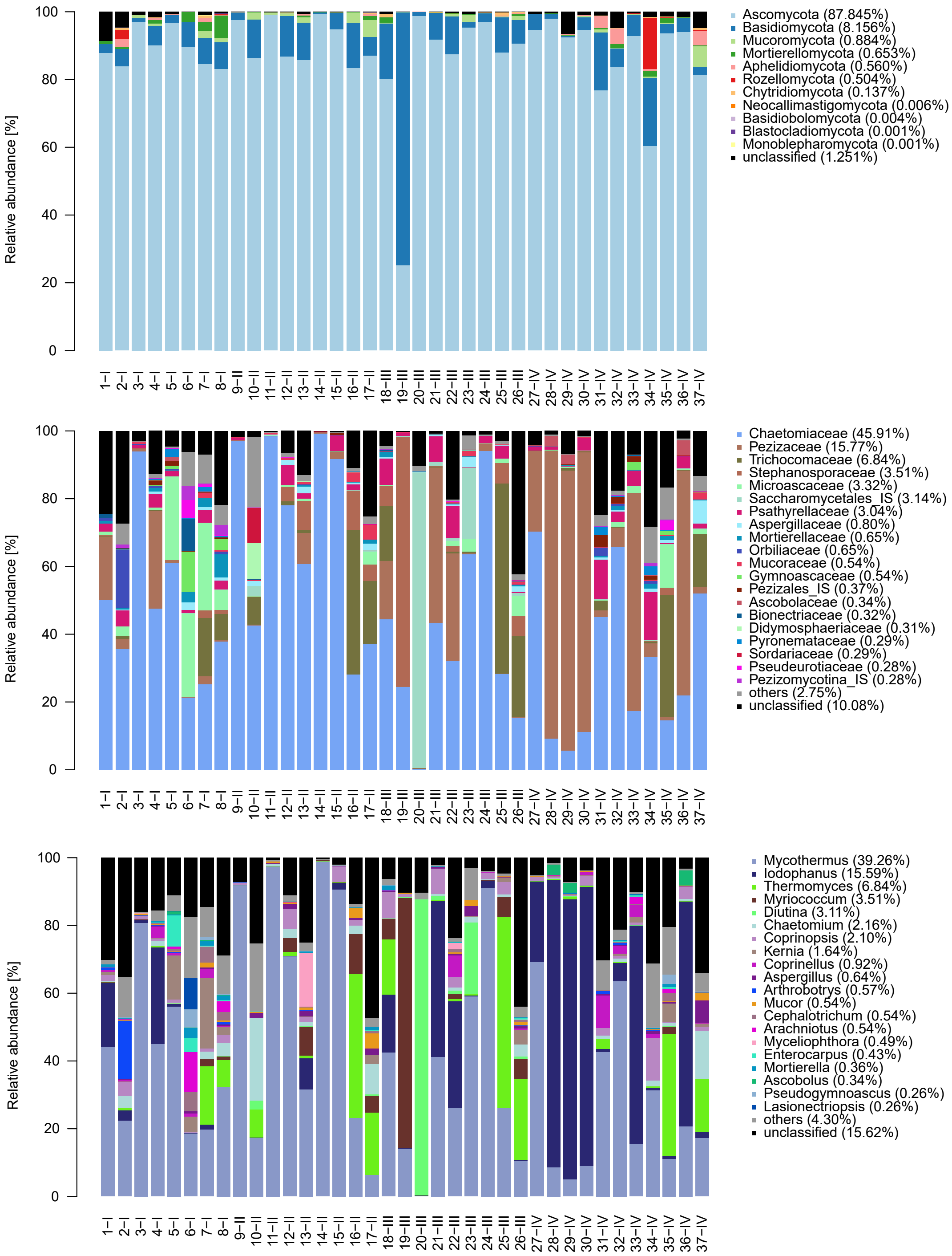

Figure S13: **Taxonomic composition of the fungal communities of the 37 composts** at (A) phylum, (B) family and (C) genus level. Taxonomic composition was calculated based on rarefied read numbers and averaged for the sequencing replicates (n=4).

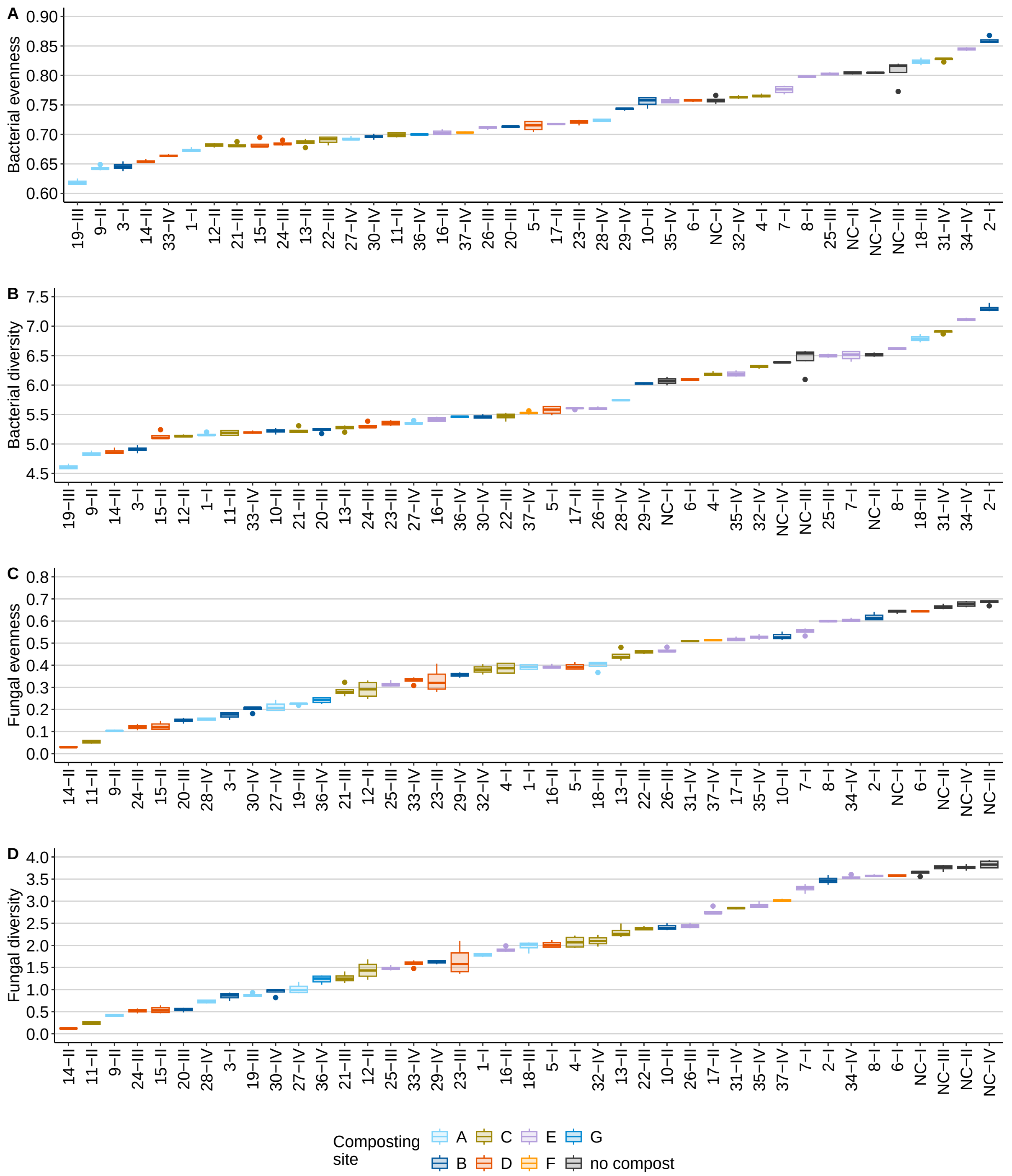

Figure S14: **Shannon eveness and Shannon diversity index of the bacterial communities and fungal communities**. Boxplots display (A) bacterial eveness, (B) bacterial diversity, (C) fungal evenness and (D) fungal diversity of the 37 composts (1-37) and the no-compost control (NC) of the four compost sets (I-IV) ordered by their means (n =4). The boxes represent the interquartile range (IQR, 25th to 75th percentile), the horizontal line within the box the median and the whiskers shows the minima and maxima of the data up to 1.5 x IQR. Outliers are shown as individual data points. Boxplots are ordered by mean.

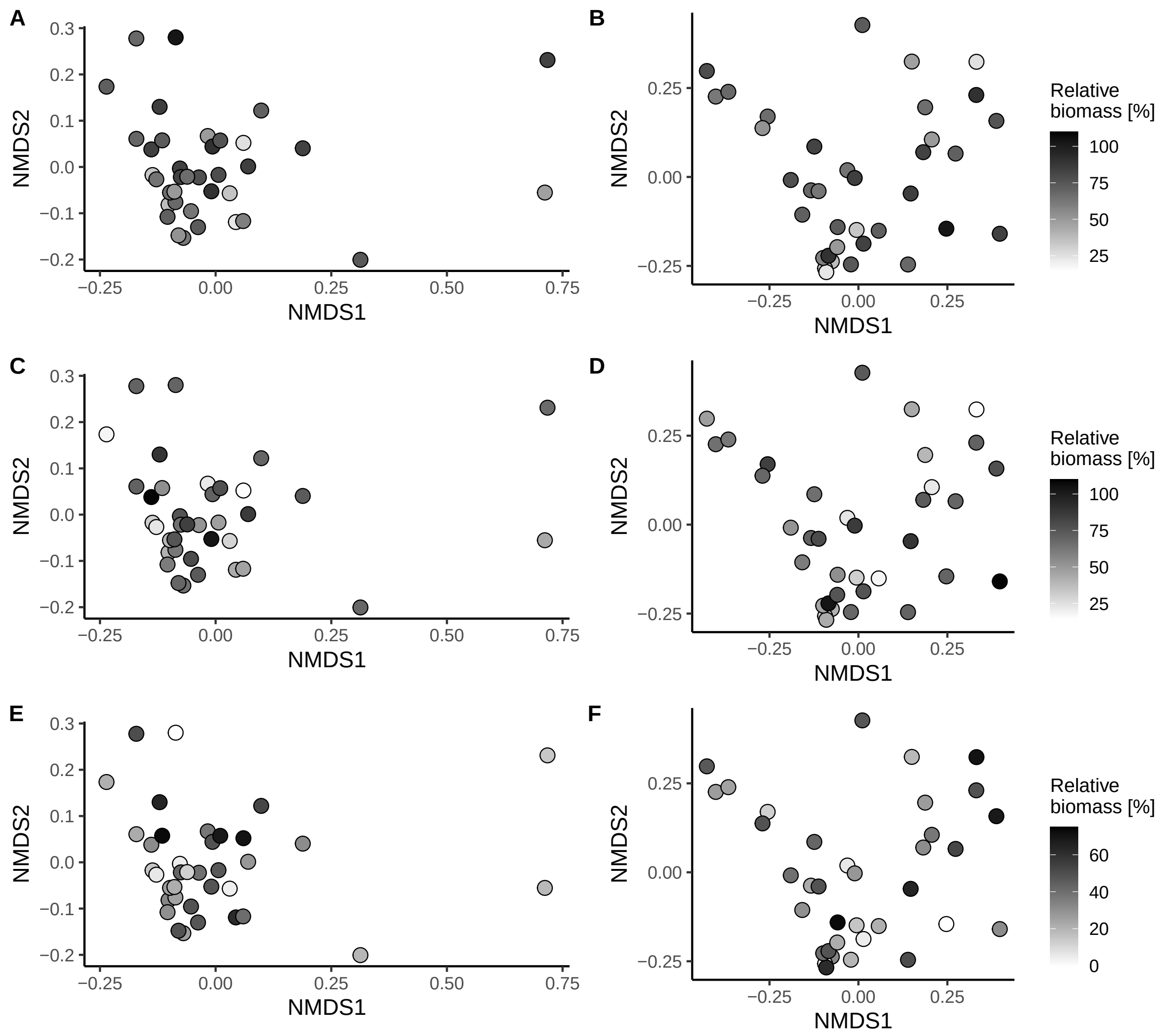

Figure S15: **(A, C, E) bacterial and (B, D, F) fungal community structure colored by disease-suppressive activity.** Community structure is based on the mean Bray-Curtis dissimilarity of four replicates for 37 composts for bacterial communities and 36 composts for fungal communities (a plot including the here excluded compost 20 is shown in Supplementary Figure S11). The ordination plot is colored by the disease-suppressive activity of the composts in (A, B) the cress-*G. ultimum* system, (C, D) the cucumber-*G. ultimum* system and (E, F) the cucumber-*R. solani* system.

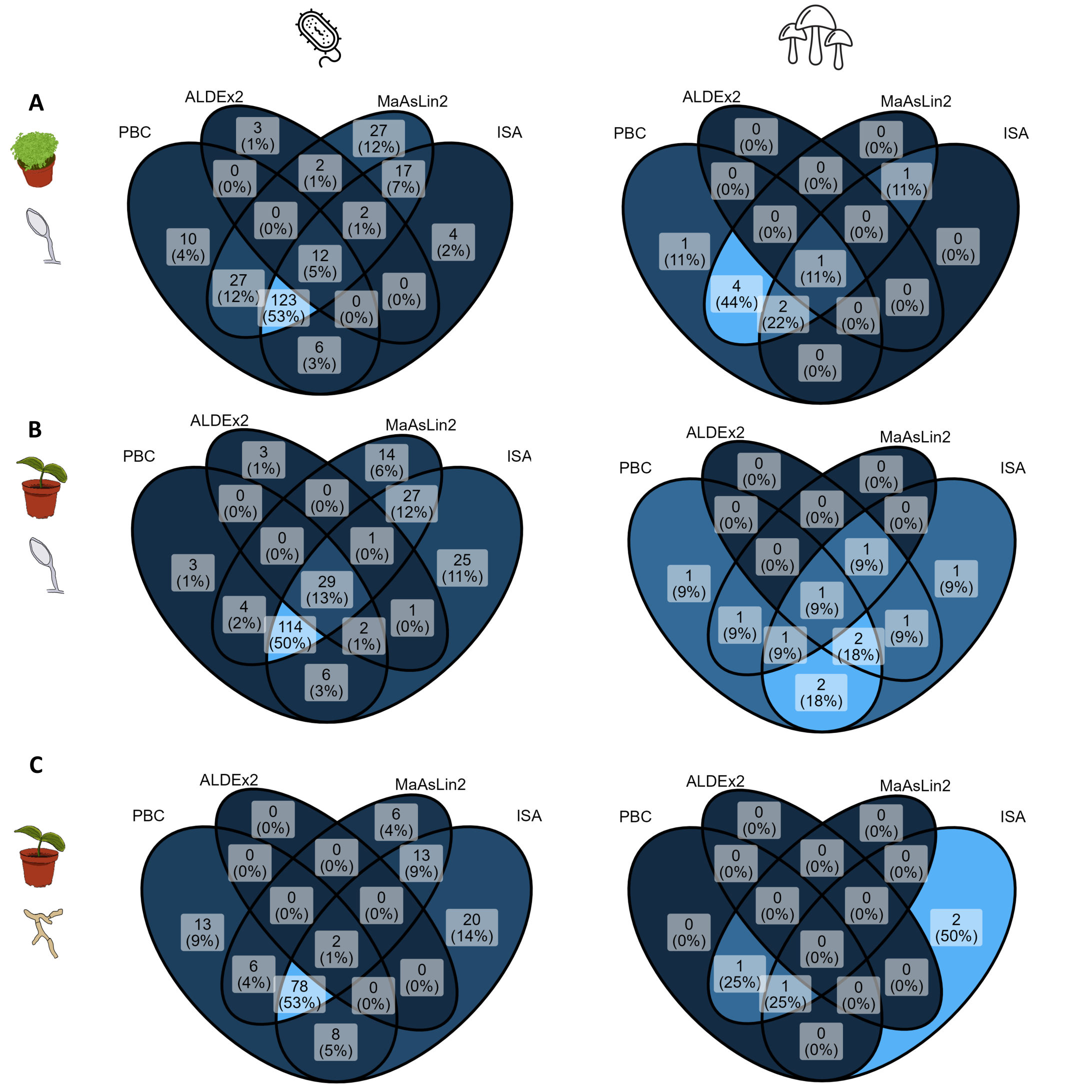

Figure S16: **Number of ASVs indicative of disease-suppressive composts identified by different statistical methods in the three plant-pathogen systems (A) cress-*G. ultimum*, (B) cucumber-*G. ultimum*, (C) and cucumber-*R. solani*.** The left column shows results for bacteria, while the right column shows results for fungi. Association tests were performed by comparing ASVs between the nine most suppressive and nine least suppressive composts for each plant-pathogen system. Only ASVs present in at least six out of the 18 selected composts were considered. ASVs were considered indicative if they were statistically significantly associated with the most suppressive composts ($p < 0.05$) in at least three out of four statistical methods (Point-biserial correlation (PBC), Indicator species analysis (ISA), MaAsLin2, ALDEx2). Venn diagrams were constructed using the *ggVennDiagram* function from the same-named R package (Gao and Dusa, 2024).

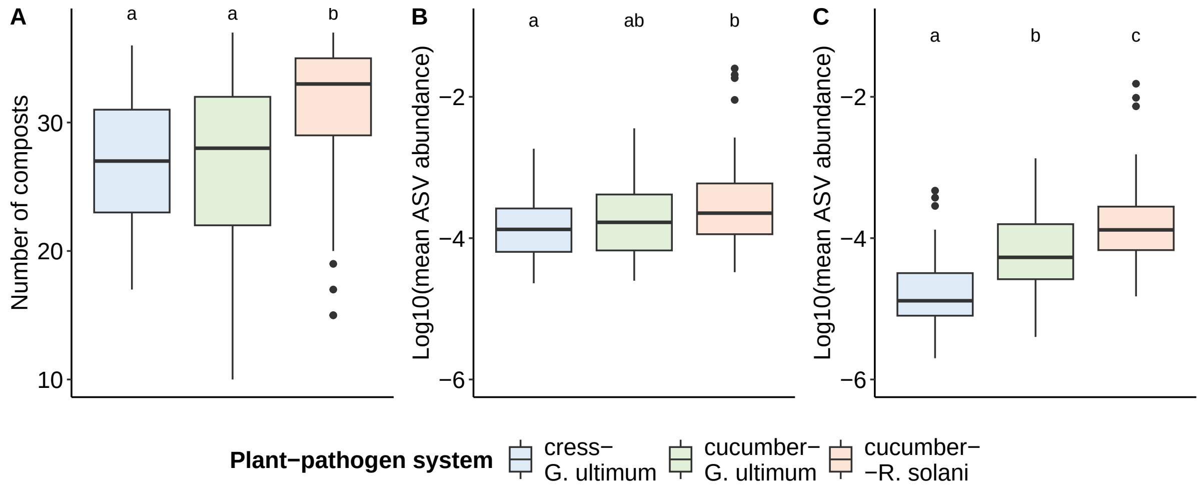

Figure S17: **Prevalence of indicative ASVs**. Boxplots displays: (A) the number of composts the indicative ASVs were detected, (B) their mean relative abundance across the disease-suppressive composts, (C) their mean relative abundance across non-suppressive composts for each plant-pathogen system (137, 146 and 80 ASVs, respectively). For plotting the relative abundances were log10 transformed. Suppressive and non-suppressive composts for each plant-pathogen system are identified in Figure 1. Statistical significance was assessed using Kruskal-Wallis tests followed by pairwise Wilcox tests: (A) Chi-squared = 47.4, p <0.001; (B) Chi-squared = 12.9, p = 0.002; and (C) Chi-squared = 136.4, p <0.001 (C).

Supplementary Tables

Table S1: **Overview composting process for the different composting sites.** During the thermophilic phase of the composting process, all composting companies turned the composting piles regularly, monitored the temperature development, and adjusted the humidity by watering the compost pile when necessary. Triangular compost piles were additionally covered with a compost fleece to control humidity in the compost pile.

Site	Composting process	Feedstock composition
A	Tabular piles with turning every 1-2 weeks<br>Compost is sieved and stored covered<br>Storage is turned manually once a week to keep aerated	10-45% green waste from the gardener<br>10-40% leaves<br>25-40% hay/grass<br>5-15% variable inputs: soil, mature compost, digestate (sieve residue)
B	Tabular piles with turning every 1-2 weeks<br>Compost is sieved and stored covered (for gardening) or uncovered (agricultural composts: 2, 10, 29)<br>Covered storage is turned manually once a week to keep aerated	0-20% green waste<br>0-10% leaves<br>60-80% digestate sieve residue<br>5-20% variable inputs: leaves, manure, digestate, cellulose, paper mill waste
C	Small triangle piles with 2-3 turnings per week<br>Maturation in large piles, covered with a fleece but not aerated<br>Sieving and covered storage	75% green waste from the gardener<br>20% mature compost<br>5% soil
D	Small triangle piles with 2-3 turnings per week<br>Compag system for two weeks for sanitation and volume reduction<br>Aeration during covered storage	55-65% municipal green waste<br>20% horse manure<br>10% soil<br>5% vegetable waste<br>0-10% mature compost
E	Small triangle piles on field edge<br>Turning only a few times<br>Sieving and storage in small covered piles	100% green waste from farm area
F	Small triangle piles with 1-2 turnings per week<br>Sieving and storage in small covered piles	100% municipal green waste
G	Tabular piles with turning every 1-2 weeks<br>Sieving and storage in small covered piles<br>Storage is turned manually once a week to keep aerated	25-30% woody green waste<br>40% leaves<br>20-25% green waste

Table S2: **Compost characteristics of the 40 composts.**

ID	age	DS	WHC	pH	sal	OD_{550}	NO_2^--N	NO_3^--N	NH_4^+-N	N_{min}	NO_3^--N/N_{min}	PO_4^{3-}-P	N_{tot}	C_{org}	C_{org}/N_{tot}	basal	FDA
Ex-1	110	53.5	2.3	7.7	9.3	0.15	0.6	3.7	3.5	7.8	0.48	9.8	1.6	24.7	15.3	14.8	9.2
1	179	59.9	1.8	7.5	11.8	0.32	0.5	181.8	1.0	183.3	0.99	12.6	1.9	22.0	11.9	11.8	7.7
2	181	32.6	2.8	7.3	8.3	0.39	0.4	416.8	0.0	417.2	1.00	42.5	2.2	25.6	11.6	10.0	13.6
3	213	48.6	2.5	7.3	12.3	0.23	1.3	173.6	2.2	177.1	0.98	15.9	1.9	27.7	14.6	15.7	8.9
Ex-1	70	56.0	2.1	8.1	7.3	0.14	0.2	6.7	0.7	7.6	0.89	3.7	1.2	17.4	14.0	8.9	6.7
4	146	48.4	2.4	8.0	10.0	0.16	0.4	327.9	0.0	328.3	1.00	9.3	1.5	20.2	13.3	10.9	7.3
5	49	59.8	1.8	8.0	8.6	0.22	1.3	44.8	0.3	46.4	0.97	4.7	1.3	16.5	12.3	19.1	8.5
6	257	83.3	0.8	7.5	9.3	0.10	0.9	532.5	28.2	561.6	0.95	8.4	1.0	11.4	10.9	3.1	3.2
7	146	58.8	2.0	7.8	10.9	0.28	0.9	245.8	0.7	247.4	0.99	21.2	2.0	22.7	11.5	26.3	15.6
8	280	60.5	1.7	7.4	10.9	0.09	1.2	1097.4	0.0	1098.6	1.00	23.5	1.6	17.4	10.7	6.4	8.3
Ex-2	125	56.4	2.1	7.7	10.2	0.16	4.4	71.1	11.4	86.9	0.82	11.5	1.8	25.6	14.6	11.3	8.8
9	173	57.5	2.0	7.6	10.9	0.17	4.7	65.0	10.1	79.8	0.81	12.8	1.8	24.5	14.0	10.3	8.3
10	108	55.8	2.1	7.8	14.5	0.78	0.2	0.9	5.1	6.2	0.14	44.5	2.0	26.5	13.2	NA	26.4
11	34	61.8	1.5	7.8	7.9	0.18	79.5	158.8	71.6	309.9	0.51	9.2	1.2	15.4	12.5	10.0	8.3
12	81	66.8	1.4	7.7	9.5	0.29	10.3	364.9	43.1	418.3	0.87	11.5	1.2	15.2	12.4	6.1	9.7
13	133	62.3	1.7	7.9	9.2	0.14	14.4	194.1	39.4	247.9	0.78	8.9	1.3	16.7	13.4	9.8	8.3
14	48	74.2	1.0	7.7	6.8	0.14	44.6	220.4	3.1	268.1	0.82	11.0	0.9	10.8	11.4	7.9	8.6
15	61	75.2	0.9	7.7	6.0	0.13	0.5	107.1	0.5	108.1	0.99	9.3	0.8	8.6	11.2	8.1	9.0
16	90	74.0	1.5	7.7	8.8	0.17	4.6	292.9	5.0	302.5	0.97	13.4	1.6	20.9	13.0	8.2	15.6
17	167	69.9	1.8	7.7	14.1	0.25	28.9	954.1	4.1	987.1	0.97	17.1	2.0	21.4	10.9	9.7	15.0
18	153	52.5	1.5	7.5	9.6	0.78	1.1	191.2	2.0	194.3	0.98	14.1	1.5	20.0	13.0	11.6	2.9
19	195	65.1	1.6	7.5	10.8	0.25	1.4	135.1	109.6	246.1	0.55	13.8	1.7	23.8	13.7	7.5	4.9
20	117	47.9	2.7	8.0	18.8	1.10	0.4	1.5	17.2	19.1	0.08	56.3	2.3	33.5	14.4	NA	15.5
21	52	58.3	1.8	7.8	10.4	0.32	18.4	119.2	4.1	141.7	0.84	8.2	1.5	17.9	12.3	13.8	5.1
22	104	66.9	1.4	7.5	9.6	0.19	0.6	420.6	2.5	423.7	0.99	11.7	1.2	14.5	11.8	8.3	3.5
23	25	62.7	1.5	8.6	14.3	1.02	0.5	3.5	942.7	946.7	0.00	14.4	1.5	18.7	12.6	31.0	9.2
24	49	65.5	1.2	7.8	8.3	0.44	22.3	59.4	2.1	83.8	0.71	7.0	1.2	13.1	11.0	12.7	5.0
25	62	57.8	2.0	8.0	13.0	0.49	1.0	150.2	3.8	155.0	0.97	14.5	2.3	23.9	10.6	28.2	9.2
26	237	70.0	1.5	7.6	15.0	0.22	37.2	1068.3	139.6	1245.1	0.86	18.9	2.0	19.7	10.1	8.7	10.9
27	141	48.9	2.5	7.7	6.7	0.14	12.9	49.0	2.8	64.7	0.76	11.3	1.7	23.8	14.4	17.0	12.3
28	170	44.5	2.2	7.4	11.0	0.31	292.8	178.5	6.2	477.5	0.37	16.7	1.7	22.1	13.1	20.7	9.2
29	176	37.8	2.4	7.3	15.0	0.41	62.8	254.1	13.9	330.8	0.77	19.2	2.0	28.8	14.5	NA	17.6
30	239	37.3	2.5	7.7	14.3	0.45	29.9	537.9	5.0	572.8	0.94	19.2	2.2	29.0	13.1	20.0	12.1
31	NA	40.5	2.5	8.5	4.7	0.05	0.0	0.5	1.2	1.7	0.27	4.3	1.3	20.7	16.4	16.6	12.6
32	NA	48.7	2.1	8.6	6.1	0.14	0.6	149.0	2.8	152.4	0.98	5.1	1.4	17.5	12.6	7.7	9.5
33	216	56.1	1.6	7.9	6.3	0.10	1.9	130.2	3.6	135.7	0.96	5.4	1.3	16.9	13.2	10.3	6.1
34	164	46.3	2.4	8.4	4.7	0.11	0.6	29.2	1.5	31.3	0.93	5.9	1.5	22.2	14.4	15.5	10.8
35	213	69.0	1.4	7.8	15.0	0.13	1.2	870.3	8.9	880.4	0.99	12.8	1.9	19.6	10.6	7.1	11.4
36	129	47.0	2.1	7.6	10.1	0.48	230.9	49.8	250.8	531.5	0.09	11.5	1.7	23.2	13.4	15.8	7.1
37	196	43.5	2.5	8.3	8.8	0.23	59.9	94.5	184.5	338.9	0.28	6.6	1.9	23.0	12.4	9.0	7.9

DS = dry substance, WHC = max. waterholding capacity, sal = salinity, OD_{550} = humic soluble substances, basal = basal respiration, FDA = FDA hydrolysis. For units see Table S8

Table S3: **Number of sequences and ASVs for the different filtering steps of sequencing data.** High-quality sequences represent those retained after quality control using the bioinformatics pipeline. For the rarefied dataset, values are provided both with and without the non-compost control (NC). The final column indicates the percentage of sequences or ASVs lost relative to the preceding step. For fungi, three samples were excluded due to having fewer than 10,000 reads.

	**Step**	**NC included**	**Type**	**Total**	**Min**	**Max**	**Mean**	**Lost (%)**
**Bacteria**	High-quality sequences	yes	Sequences	19,933,068	36,402	330,947	121,543	
			ASVs	22,761	1,381	7,575	4,037	
	Bacterial sequences	yes	Sequences	19,706,220	35,918	328,121	120,160	1.1%
			ASVs	22,608	1,375	7,530	4,006	0.7%
	After normalization	yes	Sequences	5,823,664	34,741	36,758	35,510	70.4%
	(35,928 reads)		ASVs	22,269	881	5,823	2,730	1.5%
		no	Sequences	5,255,759	35,741	36,758	35,512	
			ASVs	19,129	881	5,823	2,696	
**Fungi**	High-quality sequences	yes	Sequences	23,267,141	1,583	206,235	132,200	
			ASVs	5,078	134	1,302	675	
	Fungal sequences	yes	Sequences	18,886,962	1,313	186,565	115,164	18.8%
			ASVs	2,913	93	740	371	42.6%
	After normalization	yes	Sequences	1,634,025	10,084	10,234	10,149	91.3%
	(10,211 reads)		ASVs	2,767	28	379	157	5.0%
		no	Sequences	1,472,158	10,084	10,234	10,153	
			ASVs	2,309	28	379	147	

Table S4: **Differences in disease-suppressive activity among composts and no-compost controls**. An ANOVA was conducted to compare substrates within each set. For disease suppression in set I within the cucumber-*G. ultimum* system, normality assumptions were violated; therefore, a Kruskal-Wallis test was applied instead.

**Plant-pathogen system**	**Compost set**	**Pathogen concentration**	**F / Chi-sq**	***p***
cress-*G. ultimum*	I	0.45	8.38	0.001
	II	0.45	6.26	0.001
	III	0.45	8.18	0.001
	IV	0.45	5.95	0.001
cucumber-*G. ultimum*	I	0.45	39.28	0.001
	II	0.45	2.42	0.019
	III	1.35	5.83	0.001
	IV	1.35	9.48	0.001
cucumber-*R. solani*	I	1.4	5.59	0.001
	II	0.8	7.44	0.001
	III	0.8	2.8	0.001
	IV	0.8	5.96	0.001

Table S5: **The five most common ASV classifications at the phylum, family, and genus levels for the 19,129 bacterial and 2,309 fungal ASVs identified across the 37 composts**. Among 64 bacterial phyla, 18 were present in all composts. At the family level, 75.3% of bacterial ASVs were assigned to 759 families, with 90 found in all composts, while 48.2% of bacterial ASVs were assigned to 1,582 genera, 78 of which were shared across all samples. For fungi, 3 of 11 phyla were found in all composts. At the family level, 67.7% of fungal ASVs were assigned to 197 families, with six found in all composts, and at the genus level, 57.8% of fungal ASVs were assigned to 375 genera, seven of which were present in all composts.

	N	**Phylum**	N	**Family**	N	**Genus**
**Bacteria**	5481	*Pseudomonadota*	510	*Sphingobacteriaceae*	324	*Flavobacterium*
	2309	*Bacteroidota*	453	*Sphingomonadaceae*	186	*Sphingobacterium*
	1516	*Actinomycetota*	389	*Chitinophagaceae*	122	*Acinetobacter*
	1227	*Patescibacteria*	384	*Flavobacteriaceae*	92	*Cellvibrio*
	1161	*Bacillota*	328	*Burkholderiaceae B*	84	*Gp6-AA40*
	956	Unclassified	4731	Unclassified	9902	Unclassified
**Fungi**	1231	*Ascomycota*	114	*Aspergillaceae*	54	*Mucor*
	427	*Basidiomycota*	83	*Microascaceae*	41	*Penicillium*
	106	*Chytridiomycota*	55	*Mortierellaceae*	37	*Aspergillus*
	87	*Mucoromycota*	50	*Mucoraceae*	26	*Coprinopsis*
	68	*Mortierellomycota*	67	*Chaetomiaceae*	25	*Talaromyces*
	289	Unclassified	750	Unclassified	975	Unclassified

N = Number of ASVS

Table S6: **ANOVA tables for differences in alpha diversity metrics among composts.**

**Group**	**Metric**	**F-value**	***p***
Bacteria	Richness	778.7	<0.001
	Shannon evenness	450.2	<0.001
	Shannon diversity	587.8	<0.001
Fungi	Richness	347	<0.001
	Shannon evenness	448	<0.001
	Shannon diversity	543.9	<0.001

Table S7: **Relationships between compost properties and microbial activity with the bacterial and fungal communities.** The analysis was based on all 37 composts, except for basal respiration (n = 34) and compost age (n = 35). Statistics: Spearman's rank-sum correlation was used to assess the relationship between alpha diversity metrics and compost properties, while PERMANOVA was applied to evaluate beta diversity and compost properties. P-values were adjusted (p-adj) for multiple testing for each community metric separately using the Benjamini & Hochberg correction.

		**Richness**			**Shannon Evenness**			**Shannon Diversity**			**Community Structure**			
**Bacteria**		ρ	p	p-adj	ρ	p	p-adj	ρ	p	p-adj	R^2 [%]	F	p	p-adj
**Compost properties**	Compost age	0.48	**	*	0.13	ns	ns	0.30	.	ns	6.2	2.18	*	*
	Dry substance	-0.35	*	ns	-0.34	*	ns	-0.34	*	ns	6.8	2.54	**	*
	WHC	0.23	ns	ns	0.30	.	ns	0.27	ns	ns	6.4	2.41	**	*
	pH	-0.03	ns	ns	0.20	ns	ns	0.11	ns	ns	4.6	1.71	.	.
	Salinity	-0.14	ns	ns	0.03	ns	ns	-0.04	ns	ns	7.5	2.84	**	*
	OD_{550}	-0.25	ns	ns	0.01	ns	ns	-0.10	ns	ns	12.2	4.87	**	**
	NO_2^--N	-0.15	ns	ns	-0.41	*	ns	-0.25	ns	ns	5.2	1.93	.	.
	NO_3^--N	0.35	*	ns	0.09	ns	ns	0.25	ns	ns	6.1	2.28	*	*
	NH_4^+-N	-0.43	**	.	-0.29	.	ns	-0.35	*	ns	5.2	1.91	.	.
	N_{min}	0.21	ns	ns	0.11	ns	ns	0.21	ns	ns	4.3	1.57	.	.
	NO_3^--N/N_{min}	0.47	**	*	0.20	ns	ns	0.33	*	ns	8.3	3.16	**	**
	PO_4^{3-}-P	-0.03	ns	ns	0.17	ns	ns	0.07	ns	ns	11.3	4.46	**	**
	N_{tot}	0.15	ns	ns	0.25	ns	ns	0.21	ns	ns	6.1	2.27	**	*
	C_{org}	0.07	ns	ns	0.16	ns	ns	0.10	ns	ns	5.9	2.18	*	*
	C_{org}/N_{tot}	-0.17	ns	ns	-0.14	ns	ns	-0.19	ns	ns	5.9	2.19	**	*
**Microbial activity**	Basal respiration	0.07	ns	ns	0.18	ns	ns	0.15	ns	ns	7.4	2.56	**	*
	FDA hydrolysis	0.07	ns	ns	0.34	*	ns	0.22	ns	ns	8.6	3.28	**	**
**Fungi**														
**Compost properties**	Compost age	0.38	*	ns	0.41	*	ns	0.42	*	ns	8.8	3.19	**	*
	Dry substance	-0.11	ns	ns	-0.01	ns	ns	-0.03	ns	ns	7.4	2.79	**	*
	WHC	0.14	ns	ns	0.06	ns	ns	0.09	ns	ns	4.6	1.69	ns	ns
	pH	0.13	ns	ns	0.03	ns	ns	0.09	ns	ns	4.3	1.56	ns	ns
	Salinity	-0.17	ns	ns	0.03	ns	ns	0.00	ns	ns	10.2	3.97	**	*
	OD_{550}	-0.38	*	ns	-0.21	ns	ns	-0.28	.	ns	5.2	1.94	.	.
	NO_2^--N	-0.23	ns	ns	-0.40	*	ns	-0.39	*	ns	7.5	2.84	**	*
	NO_3^--N	0.33	*	ns	0.31	.	ns	0.32	.	ns	9.4	3.63	**	*
	NH_4^+-N	-0.24	ns	ns	-0.21	ns	ns	-0.21	ns	ns	1.3	0.47	ns	ns
	N_{min}	0.37	*	ns	0.24	ns	ns	0.27	ns	ns	7.0	2.62	*	*
	NO_3^-/N_{min}	0.40	*	ns	0.37	*	ns	0.37	*	ns	4.4	1.62	ns	ns
	PO_4^{3-}-P	-0.12	ns	ns	0.08	ns	ns	0.02	ns	ns	8.1	3.07	**	*
	N_{tot}	0.01	ns	ns	0.18	ns	ns	0.14	ns	ns	10.5	4.12	**	*
	C_{org}	-0.12	ns	ns	0.00	ns	ns	-0.04	ns	ns	7.2	2.70	*	*
	C_{org}/N_{tot}	-0.23	ns	ns	-0.27	ns	ns	-0.27	ns	ns	6.4	2.41	*	*
**Microbial activity**	Basal respiration	-0.12	ns	ns	-0.21	ns	ns	-0.21	ns	ns	2.5	0.81	ns	ns
	FDA hydrolysis	0.09	ns	ns	0.17	ns	ns	0.15	ns	ns	5.8	2.15	*	*

Units are provided in Table S8. Significance levels are denoted as follows: ns = not significant, "." p <0.1, * p <0.05, ** p <0.01, *** p <0.001.

Table S8: **Relationship between compost sets and composting sites with physicochemical properties, microbial activities, and alpha- and beta-diversity of bacterial and fungal communities.** The analysis included all 37 composts for compost set comparisons and 35 composts collected from composting sites A–F for site-specific comparisons. Three data points were missing for basal respiration (10, 20, 29), and two were missing for compost age (31, 32). Statistics: A Kruskal-Wallis test was used for univariate analysis, while PERMANOVA was applied for multivariate analysis. *P*-values were adjusted (*p*-adj) for multiple testing separately for physicochemical properties/microbial activities and alpha diversity metrics.

		**Compost set**			**Composting site**		
		**Chi-sq**	**p**	**p-adj**	**Chi-sq**	**p**	**p-adj**
**Compost properties**	Compost age [days]	11.22	*	.	9.21	.	.
	DS [%]	14.63	**	*	15.67	**	**
	Max. WHC [a]	9.68	*	.	16.1	**	**
	pH	3.51	ns	ns	8.77	.	.
	Salinity [b]	3.58	ns	ns	12.33	*	*
	OD_{550}	8.28	*	ns	9.66	*	.
	NO_2^--N [c]	6.21	ns	ns	1.04	ns	ns
	NO_3^--N [c]	3.65	ns	ns	6.36	ns	ns
	NH_4^+-N [c]	10.7	*	.	0.36	ns	ns
	N_{min} [c]	0.37	ns	ns	3.38	ns	ns
	NO_3^-/N_{min}	12.13	**	.	4.09	ns	ns
	PO_4^{3-}-P [c]	2.81	ns	ns	22.63	***	***
	N_{tot} [%]	2.36	ns	ns	28.23	***	***
	C_{org} [%]	3.55	ns	ns	26.98	***	***
	C_{org}/N_{tot}	5.7	ns	ns	14.05	**	*
**Microbial activity**	Basal respiration [d]	4.89	ns	ns	2.34	ns	ns
	FDA hydrolysis [e]	4.95	ns	ns	15.52	**	*
**Alpha diversity**	Bacterial richness	15.20	**	**	6.68	ns	ns
	Bacterial Shannon evenness	5.53	ns	ns	7.16	ns	ns
	Bacterial Shannon diversity	10.66	*	*	8.21	ns	ns
	Fungal richness	10.82	*	*	11.20	*	.
	Fungal Shannon evenness	5.90	ns	ns	8.77	.	.
	Fungal Shannon diversity	6.87	.	ns	9.98	*	.
		**F (R^2)**	***p***	***p*-adj**	**F (R^2)**	***p***	***p*-adj**
**Multivariate analysis**	PCA physicochemical properties	2.01 (15.4%)	**	NA	3.68 (42.4%)	***	NA
	Bacteria community structure	1.55 (12.3%)	*	NA	2.72 (26.6%)	***	NA
	Fungi community structure	1.80 (14.0%)	*	NA	2.71 (26.5%)	***	NA

ns = not significant, "." p <0.1, "*" p <0.05, "**" p <0.01, "***" p <0.001,

[a] [g H_20 (g DS)$^{-1}$] , [b] [g KCl_{eq} (kg DS)$^{-1}$], KCl_{eq} = Potassium chloride-equivalent, [c] [mg (kg DS)$^{-1}$] , [d] [mg CO_2-C h^{-1}], [e] [μg FDA (min g DS)$^{-1}$]

Table S9: **Taxonomic classification of indicative ASVs.** The table provides the number of distinct ASV classifications (N) and the percentage of ASVs classified (%) at the phylum, family, and genus levels. Additionally, it highlights the three most prevalent ASV classifications at each level, specifying the number of indicative ASVs assigned to these classifications (n). Sometimes several classifications had the same number of ASVs, in which case all of them are listed.

**plant-path system**	**Phylum**			**Family**			**Genus**		
	N (%)	n	Classification	N (%]	n	Classification	N (%)	n	Classification
G. ultimum-cress	12 (96%)	49	*Pseudomonadota*	53 (71%)	9	*Rhizobiaceae*	48 (23%)	3	*Synoicihabitans*
		18	*Chloroflexota*		8	*Cyclobacteriaceae*		3	*Devosia*
		18	*Bacteroidota*		6	*Opitutaceae*		2	*Microvirga, Cellvirbio, Flavobacterium*
G. ultimum-cucumber	14 (96%)	54	*Pseudomonadota*	53 (91%)	10	*Sphingomonadaceae*	50 (37%)	4	*Flavobacterium*
		26	*Bacteroidota*		8	*Cyclobacteriaceae*		3	*W-Chloroflexi-9, VFJN01, Streptomyces, Luteimonas_D*
		17	*Actinomycetota*		6	*Xanthomonadaceae*			
R. solani-cucumber	12 (95%)	21	*Pseudomonadota*	35 (70%)	6	*Streptosproangiacaeae*	27 (35%)	4	*Aggregatillinea*
		20	*Actinomycetota*		4	*Steroidobacteraceae*		3	*Sphearobacter, Nonomuraea*
		13	*Chloroflexota*			*Thermomicrobiaceae, Aggregatilineaceae*			

Table S10: **Indicative ASVs which had a sequence match of at least 99% with one of the 75 indicative ASVs in Mayerhofer et al. (2021)**. In this study, ASVs were classified using the GTDB. In our previous project, sequences were also clustered into ASVS but referred to as ESV (exact sequence variant) and classified with the SILVA database.

ASV	ESV	plant-path	match [%]	GTDB phylum	GTDB lowest	SILVA lowest
87	1266	cr-Gu	99.3	*Pseudomonadota*	f *Steroidobacteraceae*	g *Steroidobacter*
1113	1266	cr-Gu	99.1	*Pseudomonadota*	s *Povalibacter uvarum*	g *Steroidobacter*
652	250	cr-Gu	99.3	*Pseudomonadota*	g *Devosia*	g *Devosia*
1431	3619	cr-Gu	99.6	*Chloroflexota*	f UBA6265	f JG30-KF-CM45
1710	3867	cr-Gu	99.6	*Pseudomonadota*	f *Rhizobiaceae*	o *Rhizobiales*
146	162	cuc-Gu	100.0	*Pseudomonadota*	g *Sphingopyxis*	g *Sphingopyxis*
1823	11259	cuc-Gu	99.5	*Pseudomonadota*	o *Pseudomonadales*	c *Gammaproteobacteria*
3139	11259	cuc-Gu	99.8	*Pseudomonadota*	o *Pseudomonadales*	c *Gammaproteobacteria*
2470	7931	cuc-Gu	100.0	*Pseudomonadota*	f SG8-39	o *Betaproteobacteriales*
467	64	Gu	99.8	*Bacteroidota*	s *Algoriphagus terrigena*	g *Algoriphagus*
2708	2082	Gu	99.3	*Pseudomonadota*	o *Rhizobiales*	g Pseudorhodoplanes
269	48	cuc-Rs	99.0	*Pseudomonadota*	o *Rhizobiales*	o *Rhizobiales*

cr-Gu = cress-*G. ultimum*, cuc-Gu = cucumber-*G. ultimum*, cuc-Rs = cucumber-*R. solani*,
c = class, o = order, f = family, g = genus, s = species

Table S11: **Effect of compost age and disease suppression in cress-*G. ultimum* combined on bacterial community structure of the 17 composts published by Mayerhofer et al. (2021)**. Compost age was only available for 12 of the 17 composts. Bacterial community structure was based on Bray-Curtis dissimilarities using mean relative abundance of ASVs. Dissimilarity based linear models were performed with PERMANOVA.

**Factor**	**Df**	**explained variability**	**Pseudo-F**	***p*-value**
age	1	17.6	2.1	0.038
Disease suppression	1	7.5	0.9	0.524
Residuals	9	75		
Total	11	100		

Table S12: **Genera assigned to indicative ASVs for which their presence was previously reported in composts**

cr-Gu	cuc-Gu	cuc-Rs	Genus	Literature on presence in compost
	ASV864		*Advenella*	Isolated from multiple waste compost (Tondello et al., 2022)
		ASV4	*Aggregatilinea*	(Fan et al., 2024)
		ASV3067		
		ASV23055		
		ASV23950		
ASV3229			*Agrobacterium*	Isolate from paper sludge compost (Charest et al., 2005)
ASV467	ASV467		*Algoriphagus*	Rice straw compost (Gavande et al., 2021),
				indicator maturation phase (Tortosa et al., 2021)
		ASV298	*Anseongella*	(Ji et al., 2023)
	ASV90		*Arenimonas*	Isolated from compost (Jin et al., 2012)
ASV1927	ASV1927		*Brevundimonas*	Detected in composts (Antoniou et al., 2017),
				denitrifying bacteria (Sun et al., 2019)
		ASV2570	*Caldicoprobacter*	(Che et al., 2021)
		ASV2729	*Calditerricola*	Isolated from compost (Moriya et al., 2011)
ASV2571			*Cellulosimicrobium*	(Hu et al., 2021a)
ASV231	ASV778		*Cellvibrio*	Indicator cooling phase (van Dijk et al., 2023)
ASV778				maturation phase (Li et al., 2020)
		ASV298	*Coprinellus* (fungi)	(Kurakov and Bilanenko, 2023)
ASV840			*Croceibacterium*	(Gao et al., 2024)
ASV325	ASV2820		*Devosia*	Indicator for thermophilic stage (Li et al., 2020),
ASV399				maturation phase (Blaya et al., 2016)
ASV652				
	ASV632		*Enhygromyxa*	Sheep manure composting (Cai et al., 2023)
	ASV4977			
	ASV587		*Ferrovibrio*	Isolated from compost (Wang et al., 2022b)
		ASV710	*Filomicrobium*	Isolated from compost (Li et al., 2024)
ASV780	ASV65		*Flavobacterium*	Several strains isolated from composts (Kim et al., 2012)
ASV975	ASV561			
	ASV780			
ASV739			*Glutamicibacter*	Isolated from compost (Borker et al., 2021)
	ASV1137		*Glycomyces*	Indicator for cooling phase (Zhang et al., 2021)
		ASV1249	*Hyphomicrobium*	Detected in composts (Yang et al., 2023)
	ASV9		*Kernia (fungi)*	(Zhao et al., 2023)
ASV2767	ASV58		*Luteimonas*	Isolated from compost (Young et al., 2007),
ASV903	ASV849			humification processes (Tortosa et al., 2021)
	ASV2767			
	ASV752		*Membranicola*	(Lu et al., 2022)
	ASV1498		*Methylosinus*	(Wei et al., 2016)
ASV1379			*Microvirga*	Abundant in thermophilic stage, nitrogen fixer (Parab et al., 2023)
ASV1408				
	ASV122		*Mortierella (fungi)*	Indicator for compost maturation (Kurakov and Bilanenko, 2023)

Continued on next page

cr-Gu	cuc-Gu	cuc-Rs	Genus	Literature on presence in compost
		ASV243	*Mycobacterium*	Isolated from compost (Lima-Junior et al., 2016)
ASV500	ASV500		*Nitrosomonas*	Indicator for maturation phase, nitrifier (Wang et al., 2022a)
		ASV178<br>ASV658<br>ASV1067	*Nonomuraea*	(Wu and Liu, 2016)
	ASV438		*Paracoccus*	(Tortosa et al., 2021)
	ASV1339<br>ASV1583		*Parapedobacter*	Isolated from compost (Kim et al., 2010)
ASV1781			*Phenylobacterium*	Isolated from compost (Weon et al., 2008)
ASV1113		ASV794	*Povalibacter*	Indicator for cooling phase (Zhong et al., 2020)
	ASV406		*Pseudorhizobium*	(Cai et al., 2023)
	ASV327		*Pseudoxanthomonas*	Isolated from compost (Lin et al., 2019)
	ASV2260		*Pusillimonas*	(Zainudin et al., 2020)
		ASV434	*Rhodomicrobium*	(Hu et al., 2021b)
ASV1700			*Rhizomicrobium*	Dominant in maturation phase (Bohrer et al., 2023)
		ASV76	*Scedosporium* (fungi)	Indicator for maturation phase (Gu et al., 2017)
ASV1678			*Shinella*	Isolated from compost (Vaz-Moreira et al., 2010)
		ASV10<br>ASV3223<br>ASV8464	*Sphaerobacter*	(Storey et al., 2015; Ren et al., 2023)
	ASV280		*Sphingobacterium*	Isolated from compost (Kim et al., 2006)
	ASV1563		*Sphingobium*	Isolate from compost (Vaz-Moreira et al., 2009)
	ASV146		*Sphingopyxis*	Indicator for cooling phase (Tian et al., 2013)
	ASV452<br>ASV737<br>ASV1749	ASV791	*Streptomyces*	Isolated from compost (Shen et al., 2021; Salamoni et al., 2010)
		ASV102<br>ASV105	*Thermomonospora*	Isolated from compost (Wu et al., 2019)

cr-Gu: cress-*G. ultimum*, cuc-Gu: cucumber-*G. ultimum*, cuc-RS: cucumber-*R. solani*

Table S13: **Genera assigned to indicative ASVs for potential involvement in disease suppression was previously reported.**

cr-Gu	cuc-Gu	cuc-Rs	Genus	Literature search results
	ASV864		*Advenella*	Low abundant member of suppressive syncom against root rot (Li et al., 2021)
ASV3229			*Agrobacterium*	Control of crown call (Ryder et al., 1991), disease-suppressive compost isolate (Charest et al., 2005)
ASV467	ASV467		*Algoriphagus*	Enriched in rhizosphere suppressive of bacterial wilt (Zheng et al., 2024)
	ASV90		*Arenimonas*	Indicator for suppressive soils (Ou et al., 2019)
ASV1927	ASV1927		*Brevundimonas*	Control of *Fusarium redolens* in chickpea (Bekkar and Zaim, 2024)
ASV231 ASV778	ASV778		*Cellvibrio*	Associated with bacterial wilt decrease (Messiha et al., 2023)
ASV427			*Chthoniobacter*	Consortia suppressive of *Fusarium oxysporum* (Kudjordjie et al., 2023)
ASV325 ASV399 ASV652	ASV2820		*Devosia*	Responsible for suppressive conditions in rhizosphere (Liu et al., 2024)
ASV780 ASV975	ASV65 ASV561 ASV780		*Flavobacterium*	Part of consortium suppression of *R. solani* (Carrión et al., 2019)
ASV739			*Glutamicibacter*	Isolate from compost with plant beneficial properties (Borker et al., 2021)
	ASV1137		*Glycomyces*	Antifungal properties (Ting et al., 2010)
		ASV1249	*Hyphomicrobium*	Enriched under suppressive conditions (Cao et al., 2024)
ASV2767 ASV903	ASV58 ASV849 ASV2767		*Luteimonas*	Enriched in suppressive composts (Scotti et al., 2020; Hernández-Lara et al., 2022)
ASV1379 ASV1408			*Microvirga*	Enriched in *Fusarium* wilt suppressive soils (Siegel-Hertz et al., 2018)
	ASV122		*Mortierella* (Fungi)	Associated with soils suppressive to *Fusarium* wilt (Khatri et al., 2023; De Corato et al., 2019; Xiong et al., 2017), enriched in soil suppressive of *G. ultimum* (Kurm et al., 2023)
		ASV243	*Mycobacterium*	Enriched in community suppressive of *Pythium aphanidermatum* in cucumber (Postma et al., 2005), production of siderophores (Meena et al., 2017)
ASV500	ASV500		*Nitrosomonas*	Enriched under suppressive conditions (Shen et al., 2019)
	ASV3060		*Novosphingobium*	Enriched under suppressive conditions (Deng et al., 2022)
ASV1781			*Phenylobacterium*	Associated with bacterial wilt control (Ding et al., 2023)
ASV1113		ASV794	*Povalibacter*	Enriched in *Fusarium oxysporum* suppressive soils (Ou et al., 2019)
	ASV327		*Pseudoxanthomonas*	Significantly associated with *Fusarium* wilt incidence (Wen et al., 2023)

Continued on next page

cressGu	cucGu	cucRs	Genus	Disease Suppression
ASV1700			*Rhizomicrobium*	Enriched in soil suppressive of *Fusarium* wilt (Lv et al., 2023)
ASV1678			*Shinella*	Enriched by biochar application leading to *Fusarium* suppression (Jaiswal et al., 2017)
ASV1513	ASV1513	ASV3191	*Sinorhizobium*	Enriched under suppressive conditions (Xue et al., 2015)
	ASV280		*Sphingobacterium*	Suppression of fungal pathogens (Xu et al., 2020), suppression of *Fusarium* (Matsuda et al., 2001)
	ASV1563		*Sphingobium*	Associated with bacterial wilt control (Ding et al., 2023)
	ASV146		*Sphingopyxis*	Antagonist of *Verticillium* pathogen (Snelders et al., 2020), part of a suppressive consortium (Fujiwara et al., 2016), enriched on healthy plants (Gu et al., 2022)
	ASV452<br>ASV737<br>ASV1749	ASV791	*Streptomyces*	Suppresses tomato wilt (Shen et al., 2021; Salamoni et al., 2010), antimicrobial properties (Kinkel et al., 2012), suppression of *R. solani* in pepper plants (Wang et al., 2015)
ASV2839			*Telluria*	Potential root-knot nematode control (Oka, 2010)

cr-Gu = cress-*G. ultimum*, cuc-Gu = *G. ultimum*-cucumber, cuc-Rs = cucumber-*R. solani*

Table S14: **Fungal ASVs indicative of the nine most suppressive composts** compared to the nine least suppressive. ASVs were ordered by increasing ranksum for each plant-pathogen system.

System	ASV	PBC	ISA	MAS	ALX	Rank [a]	n[b]	ρ	p	Phylum	Lowest taxonomy
cr-Gu	116	0.4	0.87	0.022	NA	5	22	0.34	0.037	*Ascomycota*	*f Pezizales IS*
cr-Gu	127	0.44	0.88	0.022	NA	3	20	0.42	0.009	*Ascomycota*	*f Ascodesmidaceae*
cr-Gu	185	0.31	0.81	0.014	NA	9	17	0.32	0.055	*Ascomycota*	*f Pezizales IS*
cuc-Gu	122	0.41	0.88	0.053	0.85	7	16	0.50	0.002	*Mortierellomycota*	*s Mortierella yunnanensis*
cuc-Gu	9	NA	0.94	0.115	0.64	11	34	0.37	0.025	*Ascomycota*	*g Kernia*
cuc-Gu	29	0.37	0.95	0.048	NA	11	29	0.39	0.016	*Basidiomycota*	*g Coprinellus*
cuc-Gu	45	0.37	0.74	NA	0.68	13	17	0.31	0.060	*Ascomycota*	*p Ascomycota*
cuc-Gu	76	0.36	0.81	NA	0.61	16	14	0.41	0.013	*Ascomycota*	*s Scedosporium prolificans*
cuc-Rs	64	0.48	0.88	0.06	NA	1	24	0.43	0.007	*Ascomycota*	*o Microascales*

[a] Rank sum: indicative ASVs were ranked based on the effect size for all four statistical methods (PBC, ISA, MAS, ALX) and then the ranks were summed up. [b] Number of composts the ASVs was detected. PBC = Point Biserial Correlation, ISA = Indicator Species Analysis, MAS = MaAsLins, ALX = ALDEx2. cu-Gu = cress-*G. ultimum*, cuc-Gu = cucumber-*G. ultimum*, cuc-Rs = cucumber-*R. solani*.

Table S15: **Bacterial ASVs indicative of the nine most suppressive composts in the cress-*G. ultimum* system.** For abbreviations see Table S14

ASV	PBC	ISA	MAS	ALX	Rank	n	rho	p	Phylum	Lowest taxonomy
618	0.6	0.92	0.017	NA	62	32	0.48	0.003	*Planctomycetota*	*f Lacipirellulaceae*
594	0.69	0.93	0.012	NA	71	29	0.45	0.006	*Planctomycetota*	*f Tepidisphaeraceae*
242	0.56	0.91	0.022	NA	84	30	0.50	0.002	*Acidobacteriota*	*o Vicinamibacterales*
622	0.57	0.92	0.013	NA	87	28	0.55	0.000	*Gemmatimonadota*	*g Palsa-1233*
198	0.56	0.91	0.019	NA	88	29	0.45	0.005	unclassified	*unclassified*
543	0.53	0.92	0.018	0.59	89	22	0.54	0.001	unclassified	*unclassified*
135	0.56	0.9	0.024	NA	92	30	0.47	0.003	*Acidobacteriota*	*g OLB17*
922	0.64	0.92	0.01	NA	93	23	0.42	0.009	*Pseudomonadota*	*g Sphingomicrobium*
1257	0.56	0.94	0.01	NA	101	20	0.58	0.000	*Planctomycetota*	*o Phycisphaerales*
94	0.53	0.91	0.03	NA	102	33	0.47	0.004	*Chloroflexota*	*f Phototrophicaceae*
1927	0.53	0.96	0.01	0.69	104	28	0.50	0.002	*Pseudomonadota*	*s Brevundimonas basaltis*
87	0.5	0.92	0.028	NA	105	36	0.38	0.022	*Pseudomonadota*	*f Steroidobacteraceae*
2750	0.6	0.92	0.009	NA	113	25	0.50	0.002	unclassified	unclassified
174	0.49	0.92	0.023	NA	114	29	0.41	0.012	unclassified	unclassified
824	0.49	0.92	0.015	0.55	120	31	0.53	0.001	*Pseudomonadota*	*f Rhizobiaceae*
702	0.6	0.88	0.013	NA	122	32	0.37	0.024	*Bacteroidota*	*f Cyclobacteriaceae*
698	0.65	0.87	0.013	NA	124	32	0.47	0.003	*Planctomycetota*	*o Pirellulales*
1379	0.52	0.95	0.01	NA	126	31	0.43	0.008	*Pseudomonadota*	*g Microvirga*
214	0.48	0.93	0.015	NA	128	29	0.54	0.001	*Patescibacteria*	s JACDBH01 sp013694995
689	0.52	0.91	0.014	0.5	129	30	0.44	0.007	*Chloroflexota*	*f Caldilineaceae*
124	0.55	0.87	0.026	NA	131	34	0.43	0.009	*Bacteroidota*	s UBA2336 sp002425185
295	0.53	0.89	0.017	NA	133	32	0.49	0.002	*Pseudomonadota*	*c Alphaproteobacteria*
107	0.56	0.86	0.02	NA	134	35	0.30	0.071	*Pseudomonadota*	s SYSU-D60015 sp003576705
500	0.5	0.91	0.016	NA	135	34	0.41	0.011	*Pseudomonadota*	*s Nitrosomonas nitrosa*
519	0.54	0.9	0.013	NA	138	29	0.32	0.052	*Bacteroidota*	*f Cyclobacteriaceae*
851	0.54	0.9	0.013	NA	138	30	0.41	0.012	*Chloroflexota*	*f UBA2979*
184	0.49	0.9	0.024	NA	138	32	0.44	0.006	*Bacteroidota*	*f Flavobacteriaceae*
234	0.55	0.87	0.019	NA	139	31	0.37	0.026	*Pseudomonadota*	*o Burkholderiales*
316	0.46	0.93	0.015	NA	139	23	0.45	0.006	*Verrucomicrobiota*	*s Nibricoccus aquaticus*
548	0.57	0.87	0.013	NA	141	32	0.39	0.016	*Acidobacteriota*	f SCN-69-37
568	0.61	0.85	0.014	0.49	144	33	0.48	0.003	*Planctomycetota*	*g Lacipirellula*
998	0.56	0.89	0.01	NA	147	33	0.32	0.053	*Acidobacteriota*	f SCN-69-37
382	0.44	0.95	0.014	NA	148	35	0.33	0.046	*Pseudomonadota*	*f Devosiaceae*
427	0.46	0.95	0.012	NA	149	30	0.40	0.015	*Verrucomicrobiota*	*g Chthoniobacter*
789	0.53	0.92	0.009	NA	151	23	0.45	0.006	*Chloroflexota*	*p Chloroflexota*
2571	0.6	0.91	0.007	NA	156	24	0.36	0.028	*Actinomycetota*	*g Cellulosimicrobium*
294	0.46	0.91	0.016	NA	158	31	0.38	0.021	*Chloroflexota*	*f Caldilineaceae*
396	0.55	0.86	0.015	NA	159	33	0.36	0.029	*Pseudomonadota*	*o Burkholderiales*
2552	0.55	0.92	0.007	0.54	159	19	0.40	0.015	*Bacteroidota*	*c Bacteroidia*
571	0.4	0.98	0.014	NA	161	26	0.37	0.024	*Chloroflexota*	*f Roseiflexaceae*
840	0.51	0.89	0.013	NA	163	29	0.38	0.019	*Pseudomonadota*	*s Croceibacterium sp001897135*
325	0.52	0.86	0.02	NA	164	25	0.41	0.012	*Pseudomonadota*	*g Devosia*

Continued on next page

ASV	PBC	ISA	MAS	ALX	Rank	n	rho	p	Phylum	Lowest taxonomy
1301	0.44	0.95	0.011	NA	166	30	0.44	0.007	*Bacteroidota*	*c Bacteroidia*
1678	0.46	0.95	0.009	NA	173	35	0.45	0.005	*Pseudomonadota*	*s Shinella granuli*
517	0.35	0.93	0.017	NA	173	31	0.43	0.007	*Patescibacteria*	s J137 sp003694175
1566	0.43	0.94	0.011	NA	175	24	0.56	0.000	*Pseudomonadota*	s SG8-41 sp023266245
739	0.45	0.94	0.01	NA	176	34	0.38	0.020	*Actinomycetota*	*s Glutamicibacter nicotianae*
1080	0.59	0.89	0.007	NA	178	20	0.47	0.004	*Pseudomonadota*	*f Sphingomonadaceae*
1431	0.54	0.89	0.009	NA	179	26	0.41	0.012	*Chloroflexota*	f UBA6265
1113	0.35	0.93	0.015	NA	180	24	0.52	0.001	*Pseudomonadota*	*s Povalibacter uvarum*
268	0.47	0.88	0.016	NA	181	32	0.41	0.012	*Bacteroidota*	*f Cyclobacteriaceae*
467	0.36	0.92	0.016	NA	181	28	0.48	0.002	*Bacteroidota*	*s Algoriphagus terrigena*
735	0.38	0.93	0.013	NA	184	25	0.49	0.002	*unclassified*	unclassified
399	0.42	0.94	0.01	NA	185	32	0.43	0.009	*Pseudomonadota*	*s Devosia sp024707125*
832	0.51	0.88	0.011	NA	186	28	0.36	0.029	*Pseudomonadota*	*c Gammaproteobacteria*
1253	0.59	0.87	0.008	NA	186	19	0.44	0.006	*Fibrobacterota*	*s Chersky-265 sp019509785*
717	0.52	0.87	0.012	NA	187	32	0.40	0.014	*Bacteroidota*	*f Cyclobacteriaceae*
2247	0.61	0.89	0.006	NA	188	25	0.49	0.002	*Pseudomonadota*	*o Rhizobiales*
780	0.45	0.92	0.01	NA	189	20	0.38	0.020	*Bacteroidota*	*s Flavobacterium marinum*
891	0.5	0.88	0.011	NA	190	31	0.46	0.004	*Planctomycetota*	*f Lacipirellulaceae*
1144	0.32	0.92	0.014	0.54	190	20	0.50	0.002	*Bacteroidota*	*f Cyclobacteriaceae*
974	0.62	0.84	0.009	0.43	191	29	0.36	0.030	*Chloroflexota*	s WHTK01 sp009377745
2202	0.65	0.88	0.006	NA	191	22	0.54	0.001	*Planctomycetota*	s JADLFV01 sp020849625
498	0.46	0.91	0.01	NA	193	30	0.44	0.006	*Patescibacteria*	*o Saccharimonadales*
1058	0.62	0.85	0.008	NA	198	34	0.47	0.003	*Actinomycetota*	*f Ilumatobacteraceae*
1513	0.56	0.84	0.009	0.62	200	26	0.47	0.004	*Pseudomonadota*	*s Sinorhizobium fredii*
1777	0.52	0.89	0.008	NA	205	29	0.40	0.014	*Bacteroidota*	*o Cytophagales*
1729	0.56	0.86	0.008	NA	206	22	0.48	0.003	*Planctomycetota*	*f Phycisphaeraceae*
1341	0.43	0.92	0.009	NA	209	27	0.28	0.099	*Pseudomonadota*	*f Rhizobiaceae*
231	0.36	0.88	0.02	NA	215	25	0.49	0.002	*Pseudomonadota*	*g Cellvibrio*
955	0.58	0.82	0.009	NA	215	28	0.37	0.026	*Pseudomonadota*	*f Burkholderiaceae B*
2418	0.47	0.94	0.006	NA	217	28	0.32	0.055	*Pseudomonadota*	*f Rhizobiaceae*
778	0.34	0.91	0.013	NA	217	26	0.43	0.008	*Pseudomonadota*	*g Cellvibrio*
1488	0.42	0.93	0.008	NA	218	21	0.53	0.001	*Bacteroidota*	*f Cyclobacteriaceae*
1696	0.46	0.92	0.007	NA	219	20	0.46	0.004	*Pseudomonadota*	*s Vitreimonas sp900696695*
1432	0.49	0.83	0.013	0.67	220	17	0.24	0.161	*Planctomycetota*	*f Planctomycetaceae*
744	0.56	0.83	0.009	NA	221	32	0.35	0.033	*Pseudomonadota*	f CACIAM-22H2
3229	0.55	0.9	0.005	NA	222	29	0.52	0.001	*Pseudomonadota*	*s Agrobacterium salinitolerans*
645	0.46	0.87	0.011	NA	224	30	0.30	0.070	*Pseudomonadota*	s ZC4RG30 sp003242275
1966	0.56	0.87	0.006	NA	225	29	0.43	0.007	*Chloroflexota*	*f Tepidiformaceae*
1824	0.41	0.96	0.007	NA	226	24	0.47	0.003	*Verrucomicrobiota*	*s Synoicihabitans sp023386835*
2427	0.52	0.9	0.006	NA	227	27	0.50	0.002	*Hydrogenedentota*	f SLHB01
2811	0.62	0.84	0.006	0.52	231	23	0.49	0.002	*Pseudomonadota*	*f Sphingomonadaceae*
975	0.48	0.86	0.01	NA	231	24	0.38	0.021	*Bacteroidota*	*s Flavobacterium marinum*
1145	0.57	0.84	0.007	NA	233	24	0.43	0.007	*Pseudomonadota*	*o Burkholderiales*
1534	0.46	0.89	0.008	NA	239	30	0.54	0.001	*Chloroflexota*	s W-Chloroflexi-9 sp023228825
1392	0.5	0.85	0.009	NA	243	25	0.36	0.028	*Acidobacteriota*	*g Gp6-AA40*

Continued on next page

ASV	PBC	ISA	MAS	ALX	Rank	n	rho	p	Phylum	Lowest taxonomy
914	0.4	0.88	0.011	NA	243	31	0.36	0.029	*Chloroflexota*	s QEUY01 sp003577365
1157	0.36	0.91	0.009	NA	244	27	0.41	0.011	*Pseudomonadota*	*f Rhizobiaceae*
1261	0.54	0.84	0.008	NA	245	31	0.43	0.009	*Pseudomonadota*	*c Alphaproteobacteria*
937	NA	0.89	0.01	0.64	246	24	0.31	0.063	*Chloroflexota*	s UBA6265 sp023957015
1781	0.56	0.82	0.007	NA	251	27	0.44	0.007	*Pseudomonadota*	*s Phenylobacterium sp024699985*
260	0.35	0.93	0.007	NA	252	29	0.29	0.079	*Actinomycetota*	*f Micromonosporaceae*
3097	0.57	0.85	0.005	NA	253	30	0.45	0.005	*Planctomycetota*	*o Pirellulales*
1075	0.39	0.91	0.008	NA	255	27	0.27	0.103	*Chloroflexota*	*f Chloroflexaceae*
4088	0.64	0.84	0.004	NA	257	31	0.35	0.032	*Chloroflexota*	s SHYQ01 sp903917615
2096	NA	0.9	0.009	0.53	258	18	0.29	0.078	*Planctomycetota*	*f Planctomycetaceae*
1277	0.55	0.85	0.006	NA	258	30	0.33	0.049	*Verrucomicrobiota*	s Opi-474 sp903830425
2602	0.46	0.88	0.007	0.49	259	23	0.41	0.012	*Bacteroidota*	*o Rhodothermales*
1264	0.5	0.81	0.01	NA	262	22	0.41	0.011	*Verrucomicrobiota*	*f Opitutaceae*
1134	0.42	0.86	0.01	NA	262	25	0.45	0.005	*Pseudomonadota*	*f Devosiaceae*
403	0.4	0.86	0.011	NA	264	23	0.49	0.002	*Bacteroidota*	*f Chitinophagaceae*
592	0.42	0.87	0.009	NA	265	23	0.45	0.005	*Pseudomonadota*	g CADEED01
4401	0.54	0.85	0.006	NA	265	17	0.50	0.002	*Pseudomonadota*	*o Rhizobiales*
3041	0.49	0.88	0.005	NA	273	24	0.41	0.012	*Verrucomicrobiota*	*s Synoicihabitans sp023386835*
826	0.38	0.89	0.008	NA	274	27	0.27	0.108	*Bacteroidota*	*s Kaistella haifensis*
2741	0.57	0.82	0.005	NA	274	31	0.39	0.018	*Actinomycetota*	*f Ilumatobacteraceae*
2488	0.57	0.82	0.005	NA	274	21	0.41	0.011	*Actinomycetota*	*f Ilumatobacteraceae*
2210	0.46	0.88	0.006	NA	278	27	0.32	0.051	*Chloroflexota*	*p Chloroflexota*
1408	0.4	0.88	0.008	NA	279	32	0.32	0.055	*Pseudomonadota*	*g Microvirga*
1198	0.55	0.85	0.004	NA	281	27	0.37	0.026	*Gemmatimonadota*	*c Gemmatimonadetes*
1046	0.48	0.85	0.007	NA	283	31	0.31	0.061	unclassified	unclassified
784	0.52	0.79	0.008	NA	284	22	0.33	0.047	*Myxococcota*	*f Polyangiaceae*
2193	0.47	0.84	0.008	NA	285	18	0.48	0.003	*Pseudomonadota*	*f Rhizobiaceae*
1291	0.41	0.87	0.008	NA	287	22	0.49	0.002	*Bacteroidota*	*f Chitinophagaceae*
1700	0.56	0.81	0.005	NA	291	20	0.37	0.024	*Pseudomonadota*	*g Rhizomicrobium*
652	0.37	0.86	0.009	NA	292	23	0.29	0.079	*Pseudomonadota*	*g Devosia*
2887	0.48	0.84	0.007	NA	292	20	0.38	0.020	*Gemmatimonadota*	*o Longimicrobiales*
2767	0.49	0.82	0.007	NA	297	22	0.37	0.025	*Pseudomonadota*	*s Luteimonas D colneyensis*
2134	0.46	0.86	0.006	NA	299	31	0.38	0.021	*Actinomycetota*	s SHVJ01 sp016870355
2309	0.58	0.78	0.004	NA	300	25	0.29	0.086	*Actinomycetota*	*o Acidimicrobiales*
2238	0.42	0.86	0.007	NA	303	21	0.51	0.001	*Pseudomonadota*	*c Gammaproteobacteria*
1532	0.45	0.81	0.009	NA	309	18	0.48	0.003	*Patescibacteria*	o UBA9983 A
1710	0.47	0.82	0.007	NA	310	19	0.44	0.007	*Pseudomonadota*	*f Rhizobiaceae*
3127	0.53	0.82	0.004	NA	314	30	0.30	0.070	*Chloroflexota*	*f Thermomicrobiaceae*
4699	0.51	0.82	0.005	NA	317	24	0.39	0.016	*Pseudomonadota*	s SCGC-AG-212-J23 sp005881595
903	0.35	0.81	0.011	NA	318	18	0.49	0.002	*Pseudomonadota*	*s Luteimonas B weifangensis*
2708	0.48	0.82	0.006	NA	320	27	0.41	0.011	*Pseudomonadota*	*o Rhizobiales*
1815	0.44	0.85	0.006	NA	324	25	0.33	0.046	*Myxococcota*	s JABFXX01 sp005887925
3518	0.49	0.84	0.004	NA	324	28	0.43	0.008	*Chloroflexota*	s JAIBBD01 sp019694855
4340	0.44	0.86	0.005	NA	326	25	0.40	0.014	*Actinomycetota*	*f Solirubrobacteraceae*
2839	0.51	0.82	0.004	NA	327	32	0.25	0.138	*Pseudomonadota*	*g Telluria*

Continued on next page

ASV	PBC	ISA	MAS	ALX	Rank	n	rho	p	Phylum	Lowest taxonomy
2366	0.48	0.82	0.005	NA	333	25	0.32	0.051	*Patescibacteria*	*o Saccharimonadales*
2329	0.47	0.77	0.006	NA	345	20	0.36	0.029	*Acidobacteriota*	f SCN-69-37
2600	0.42	0.8	0.007	NA	350	22	0.28	0.098	*Verrucomicrobiota*	*s Synoicihabitans sp023386835*
1463	0.42	0.79	0.007	NA	351	24	0.37	0.025	*Verrucomicrobiota*	f UBA11358
2100	0.41	0.84	0.005	NA	359	24	0.32	0.055	*Chloroflexota*	*o Thermomicrobiales*

Table S16: **Bacterial ASVs indicative of the nine most suppressive composts in the cucumber-*G. ultimum* system.** For abbreviations see Table S14

ASV	PBC	ISA	MAS	ALX	Rank	n	rho	p	Phylum	Lowest taxonomy
424	0.72	0.96	0.021	0.67	32	32	0.55	0.000	*Pseudomonadota*	*f Sphingomonadaceae*
661	0.73	0.99	0.017	0.53	38	30	0.62	0.000	*Pseudomonadota*	*f Burkholderiaceae C*
1339	0.64	0.99	0.015	0.92	39	21	0.61	0.000	*Bacteroidota*	*g Parapedobacter*
146	0.73	0.95	0.024	NA	59	35	0.50	0.002	*Pseudomonadota*	*g Sphingopyxis*
308	0.67	0.94	0.020	0.52	67	33	0.55	0.000	*Pseudomonadota*	*f Xanthomonadaceae*
1513	0.73	0.96	0.011	0.53	80	26	0.65	0.000	*Pseudomonadota*	*s Sinorhizobium fredii*
840	0.57	0.95	0.014	0.67	85	29	0.54	0.001	*Pseudomonadota*	*s Croceibacterium sp001897135*
184	0.54	0.98	0.026	NA	87	32	0.47	0.003	*Bacteroidota*	*f Flavobacteriaceae*
1432	0.57	0.92	0.016	0.84	94	17	0.50	0.002	*Planctomycetota*	*f Planctomycetaceae*
500	0.58	0.95	0.018	NA	96	34	0.47	0.003	*Pseudomonadota*	*s Nitrosomonas nitrosa*
58	0.53	0.94	0.041	0.52	100	37	0.45	0.005	*Pseudomonadota*	*s Luteimonas D sp000472505*
567	0.71	0.90	0.012	0.55	124	32	0.48	0.003	*Bacteroidota*	*s Flavipsychrobacter sp020636495*
384	0.60	0.91	0.015	NA	129	32	0.33	0.043	*Sumerlaeota*	s JAHLLQ01 sp020444065
124	0.55	0.90	0.025	NA	138	34	0.39	0.017	*Bacteroidota*	s UBA2336 sp002425185
3130	0.61	0.97	0.007	NA	145	24	0.52	0.001	*Planctomycetota*	*c Planctomycetia*
2100	0.63	0.96	0.007	NA	146	24	0.46	0.004	*Chloroflexota*	*o Thermomicrobiales*
1830	0.80	0.90	0.007	0.74	148	34	0.54	0.001	*Gemmatimonadota*	*f UBA6960*
252	0.51	0.93	0.017	NA	149	28	0.31	0.059	*Bacteroidota*	*s Edaphocola sp019739295*
2767	0.59	0.97	0.007	NA	151	22	0.49	0.002	*Pseudomonadota*	*s Luteimonas D colneyensis*
90	0.56	0.89	0.021	NA	151	36	0.36	0.027	*Pseudomonadota*	*s Arenimonas fontis*
538	0.55	0.92	0.011	NA	154	31	0.28	0.099	*Pseudomonadota*	*f Steroidobacteraceae*
632	0.62	0.89	0.013	0.49	156	32	0.41	0.012	*Myxococcota*	*g Enhygromyxa*
3408	0.71	0.92	0.007	0.48	157	22	0.49	0.002	*Pseudomonadota*	*c Gammaproteobacteria*
65	0.49	0.92	0.022	NA	158	33	0.41	0.012	*Bacteroidota*	*s Flavobacterium marinum*
1230	0.64	0.89	0.009	0.63	159	33	0.45	0.006	*Gemmatimonadota*	*f UBA6960*
749	0.41	0.98	0.019	0.54	160	26	0.36	0.029	*Acidobacteriota*	*f SCN-69-37*
455	0.63	0.85	0.014	0.81	162	33	0.36	0.030	*Planctomycetota*	*f Planctomycetaceae*
568	0.61	0.88	0.011	0.57	163	33	0.49	0.002	*Planctomycetota*	*g Lacipirellula*
1390	0.65	0.91	0.008	NA	166	30	0.42	0.011	*Planctomycetota*	*f Lacipirellulaceae*
702	0.58	0.90	0.011	NA	167	32	0.31	0.060	*Bacteroidota*	*f Cyclobacteriaceae*
1370	0.53	0.94	0.009	NA	168	30	0.48	0.003	*Actinomycetota*	*s VFJN01 sp009694375*
388	0.47	0.93	0.020	NA	170	26	0.44	0.007	*Pseudomonadota*	s SYSU-D60014 sp003576685
569	0.60	0.87	0.011	0.60	173	33	0.38	0.021	*Gemmatimonadota*	*c Gemmatimonadetes*
457	0.39	0.98	0.020	0.49	176	31	0.44	0.007	*Bacteroidota*	*g JJ008*
2820	0.65	0.95	0.005	NA	177	23	0.51	0.001	*Pseudomonadota*	*g Devosia*
519	0.51	0.92	0.012	NA	177	29	0.33	0.047	*Bacteroidota*	*f Cyclobacteriaceae*
523	0.51	0.90	0.015	0.50	178	32	0.43	0.008	*Bacteroidota*	*f Cyclobacteriaceae*
2096	0.47	0.92	0.012	0.61	181	18	0.55	0.000	*Planctomycetota*	*f Planctomycetaceae*
849	0.53	0.92	0.009	NA	181	28	0.42	0.009	*Pseudomonadota*	*g Luteimonas D*
1527	0.56	0.93	0.007	NA	183	27	0.48	0.003	*Pseudomonadota*	*f Sphingomonadaceae*
1578	0.49	0.97	0.009	NA	184	24	0.40	0.015	*Pseudomonadota*	*f Sphingomonadaceae*
618	0.52	0.90	0.013	NA	187	32	0.39	0.018	*Planctomycetota*	*f Lacipirellulaceae*

Continued on next page

ASV	PBC	ISA	MAS	ALX	Rank	n	rho	p	Phylum	Lowest taxonomy
320	0.52	0.88	0.017	NA	191	35	0.33	0.044	*Actinomycetota*	*s SZUA-217 sp004356805*
452	0.40	0.95	0.017	NA	196	36	0.45	0.005	*Actinomycetota*	*g Streptomyces*
561	0.30	0.99	0.016	NA	197	29	0.48	0.003	*Bacteroidota*	*g Flavobacterium*
689	0.54	0.91	0.008	NA	199	30	0.42	0.009	*Chloroflexota*	*f Caldilineaceae*
1034	0.30	0.99	0.014	0.42	202	26	0.57	0.000	*Actinomycetota*	*s VFJN01 sp009694375*
467	0.31	0.99	0.014	NA	202	28	0.52	0.001	*Bacteroidota*	*s Algoriphagus terrigena*
1762	0.55	0.91	0.007	NA	204	24	0.41	0.011	*Actinomycetota*	*f Cellulomonadaceae*
2427	0.53	0.94	0.006	NA	205	27	0.37	0.024	*Hydrogenedentota*	*f SLHB01*
438	0.45	0.93	0.013	NA	205	35	0.31	0.060	*Pseudomonadota*	*s Paracoccus kondratievae*
411	0.52	0.87	0.012	0.59	208	33	0.31	0.060	*Pseudomonadota*	*c Gammaproteobacteria*
808	0.47	0.92	0.011	NA	209	26	0.31	0.061	*Gemmatimonadota*	*f Gemmatimonadaceae*
159	0.51	0.92	0.008	NA	211	26	0.32	0.055	*Pseudomonadota*	*o Pseudomonadales*
645	0.49	0.92	0.009	NA	212	30	0.36	0.028	*Pseudomonadota*	*s ZC4RG30 sp003242275*
799	0.41	0.99	0.009	NA	215	21	0.41	0.012	*Bacteroidota*	*g Flavobacterium*
721	0.45	0.93	0.011	NA	215	28	0.29	0.084	*Bacteroidota*	*g PHOS-HE28*
1498	0.59	0.90	0.006	NA	217	31	0.44	0.006	*Pseudomonadota*	*s Methylosinus sp000685825*
1301	0.42	0.98	0.009	NA	217	30	0.44	0.006	*Bacteroidota*	*c Bacteroidia*
752	0.38	0.91	0.014	0.65	219	21	0.45	0.005	*Bacteroidota*	*s Membranicola marinus A*
154	0.53	0.85	0.016	NA	219	37	0.41	0.013	*Pseudomonadota*	*f Sphingomonadaceae*
327	0.41	0.97	0.010	NA	219	31	0.26	0.114	*Pseudomonadota*	*s Pseudoxanthomonas suwonensis A*
981	0.46	0.90	0.011	0.58	221	31	0.48	0.002	*Pseudomonadota*	*o Rhizobiales*
329	0.46	0.91	0.012	NA	221	36	0.28	0.098	*Pseudomonadota*	*f Rhizobiaceae*
543	0.53	0.86	0.013	NA	222	22	0.38	0.022	*unclassified*	*unclassified*
292	0.37	0.95	0.013	NA	222	31	0.32	0.051	*Pseudomonadota*	*o Pseudomonadales*
465	0.41	0.94	0.011	NA	223	28	0.32	0.054	*Pseudomonadota*	*f Methylophilaceae*
2552	0.43	0.94	0.009	0.49	227	19	0.54	0.001	*Bacteroidota*	*c Bacteroidia*
974	0.66	0.85	0.008	NA	231	29	0.44	0.007	*Chloroflexota*	*s WHTK01 sp009377745*
232	0.41	0.90	0.017	NA	231	33	0.22	0.186	*OLB16*	*f SURF-12*
4289	0.61	0.91	0.004	NA	235	25	0.37	0.023	*Chloroflexota*	*g W-Chloroflexi-9*
3177	0.60	0.88	0.006	NA	236	22	0.42	0.010	*Deinococcota*	*g JAAYYF01*
2377	0.64	0.89	0.005	NA	236	30	0.42	0.010	*Pseudomonadota*	*f Ferrovibrionaceae*
3314	0.58	0.90	0.005	NA	237	17	0.48	0.003	*Bacteroidota*	*g PHOS-HE28*
418	0.47	0.91	0.009	NA	237	28	0.23	0.170	*Pseudomonadota*	*o Pseudomonadales*
780	0.43	0.92	0.010	NA	239	20	0.46	0.004	*Bacteroidota*	*s Flavobacterium marinum*
2887	0.50	0.90	0.008	NA	241	20	0.44	0.007	*Gemmatimonadota*	*o Longimicrobiales*
280	0.52	0.81	0.019	NA	241	20	0.35	0.033	*Bacteroidota*	*s Sphingobacterium sp900163865*
406	0.50	0.88	0.010	NA	245	34	0.27	0.107	*Pseudomonadota*	*s Pseudorhizobium flavum*
1734	0.49	0.90	0.008	NA	247	26	0.42	0.009	*Actinomycetota*	*f Ilumatobacteraceae*
2741	0.69	0.87	0.005	NA	249	31	0.46	0.005	*Actinomycetota*	*f Ilumatobacteraceae*
4916	0.54	0.85	0.007	0.60	254	13	0.48	0.003	*Planctomycetota*	*f Planctomycetaceae*
867	0.50	0.91	0.006	NA	254	21	0.43	0.007	*Sumerlaeota*	*f Sumerlaeaceae*
2031	0.38	0.96	0.008	NA	254	26	0.40	0.014	*Sumerlaeota*	*s JAIBAT01 sp019695055*
2488	0.59	0.88	0.005	NA	255	21	0.41	0.011	*Actinomycetota*	*f Ilumatobacteraceae*
737	0.40	0.88	0.017	NA	260	19	0.45	0.006	*Actinomycetota*	*g Streptomyces*
3041	0.45	0.94	0.006	NA	261	24	0.38	0.019	*Verrucomicrobiota*	*s Synoicihabitans sp023386835*

Continued on next page

**ASV**	**PBC**	**ISA**	**MAS**	**ALX**	**Rank**	**n**	**rho**	**p**	**Phylum**	**Lowest taxonomy**
998	0.53	0.88	0.006	NA	263	33	0.25	0.142	*Acidobacteriota*	f SCN-69-37
1058	0.55	0.87	0.006	NA	265	34	0.40	0.014	*Actinomycetota*	*f Ilumatobacteraceae*
1927	0.50	0.89	0.007	NA	266	28	0.43	0.009	*Pseudomonadota*	*s Brevundimonas basaltis*
3139	0.59	0.87	0.005	NA	267	17	0.38	0.022	*Pseudomonadota*	*o Pseudomonadales*
453	0.52	0.85	0.009	NA	268	30	0.35	0.034	*Planctomycetota*	*s JAGQOG01 sp020427695*
1594	0.45	0.91	0.007	NA	270	24	0.42	0.010	*Bacteroidota*	*s Flavipsychrobacter sp020636495*
3416	0.49	0.90	0.006	NA	272	24	0.44	0.007	*Actinomycetota*	*o Acidimicrobiales*
1938	0.51	0.89	0.006	NA	274	20	0.43	0.007	*Pseudomonadota*	*f Rhodobacteraceae*
161	0.47	0.91	0.006	NA	274	32	0.18	0.280	*Pseudomonadota*	*s Halopseudomonas formosensis*
778	0.41	0.91	0.008	NA	276	26	0.34	0.041	*Pseudomonadota*	*g Cellvibrio*
19318	0.56	0.87	0.005	NA	276	32	0.31	0.066	*Chloroflexota*	*c Anaerolineae*
414	0.56	0.83	0.007	NA	277	35	0.26	0.118	*Pseudomonadota*	*c Alphaproteobacteria*
1583	0.41	0.87	0.010	0.61	281	15	0.41	0.011	*Bacteroidota*	*g Parapedobacter*
784	0.40	0.89	0.011	NA	281	22	0.30	0.071	*Myxococcota*	*f Polyangiaceae*
1749	0.45	0.90	0.007	NA	282	30	0.40	0.015	*Actinomycetota*	*g Streptomyces*
797	0.44	0.90	0.007	NA	287	23	0.33	0.049	*Pseudomonadota*	*f Burkholderiaceae C*
1085	0.69	0.82	0.005	NA	287	35	0.29	0.082	*Planctomycetota*	*f Planctomycetaceae*
667	0.42	0.88	0.010	NA	288	34	0.37	0.023	*Pseudomonadota*	*f Burkholderiaceae A*
3060	0.52	0.88	0.005	NA	289	20	0.38	0.021	*Pseudomonadota*	*g Novosphingobium*
17109	0.53	0.87	0.005	NA	291	31	0.23	0.180	*unclassified*	*unclassified*
1144	0.37	0.86	0.014	NA	303	20	0.43	0.008	*Bacteroidota*	*f Cyclobacteriaceae*
2258	0.56	0.79	0.006	NA	304	22	0.36	0.030	*Gemmatimonadota*	*f UBA6960*
766	0.48	0.86	0.007	NA	309	35	0.41	0.013	*Pseudomonadota*	*f Sphingomonadaceae*
572	0.47	0.79	0.011	NA	310	13	0.41	0.012	*Actinomycetota*	*g Glycomyces*
9006	0.53	0.85	0.005	NA	311	26	0.25	0.136	unclassified	unclassified
2074	0.52	0.86	0.005	NA	312	29	0.35	0.034	*Chloroflexota*	*g W-Chloroflexi-9*
253	0.28	0.94	NA	0.74	319	21	0.55	0.000	*Actinomycetota*	*g Promicromonospora*
864	0.44	0.79	0.010	0.57	321	10	0.44	0.007	*Pseudomonadota*	*s Advenella incenata*
1823	0.51	0.84	0.006	NA	321	23	0.28	0.098	*Pseudomonadota*	*o Pseudomonadales*
1966	0.53	0.86	0.004	NA	322	29	0.33	0.043	*Chloroflexota*	*f Tepidiformaceae*
2769	0.40	0.84	0.009	0.60	324	14	0.46	0.004	*Planctomycetota*	*f Planctomycetaceae*
171	0.34	0.96	NA	0.50	325	31	0.39	0.017	*Bacteroidota*	*f Cyclobacteriaceae*
3127	0.54	0.85	0.004	NA	326	30	0.45	0.005	*Chloroflexota*	*f Thermomicrobiaceae*
26612	0.48	0.87	0.005	NA	329	21	0.30	0.073	*Bacteroidota*	*f Saprospiraceae*
1157	0.42	0.88	0.006	NA	331	27	0.41	0.011	*Pseudomonadota*	*f Rhizobiaceae*
1254	0.49	0.86	0.005	NA	333	32	0.25	0.132	*Pseudomonadota*	*f Xanthobacteraceae*
3518	0.50	0.87	0.004	NA	336	28	0.43	0.007	*Chloroflexota*	*s JAIBBD01 sp019694855*
1491	0.54	0.83	0.004	NA	338	35	0.32	0.053	*Bacillota*	*c Bacilli*
2310	0.51	0.87	0.003	NA	340	31	0.31	0.059	*Actinomycetota*	*f Microtrichaceae*
2260	0.46	0.81	0.008	NA	342	15	0.38	0.021	*Pseudomonadota*	*s Pusillimonas D thiosulfatoxidans*
1563	0.48	0.79	0.007	NA	342	18	0.38	0.022	*Pseudomonadota*	*g Sphingobium*
1806	0.43	0.88	0.005	NA	343	30	0.32	0.053	*Pseudomonadota*	*f Beijerinckiaceae*
4088	0.55	0.83	0.003	NA	344	31	0.29	0.079	*Chloroflexota*	s SHYQ01 sp903917615
1137	0.43	0.79	0.009	NA	346	12	0.42	0.010	*Actinomycetota*	*g Glycomyces*
1484	NA	0.86	0.008	0.50	349	29	0.48	0.002	*Actinomycetota*	*s VFJN01 sp009694375*

Continued on next page

ASV	PBC	ISA	MAS	ALX	Rank	n	rho	p	Phylum	Lowest taxonomy
2470	0.46	0.88	0.004	NA	350	31	0.28	0.098	*Pseudomonadota*	*f SG8-39*
1391	0.52	0.83	0.004	NA	353	26	0.24	0.152	*Pseudomonadota*	*f Methylophilaceae*
4977	0.48	0.86	0.004	NA	360	18	0.34	0.037	*Myxococcota*	*g Enhygromyxa*
1232	0.48	0.83	0.005	NA	361	32	0.16	0.342	*Acidobacteriota*	f UBA2999
587	0.48	0.82	0.005	NA	367	33	0.17	0.309	*Pseudomonadota*	*s Ferrovibrio terrae*
1488	0.39	0.86	0.006	NA	369	21	0.40	0.014	*Bacteroidota*	*f Cyclobacteriaceae*
1856	0.50	0.81	0.004	NA	377	19	0.33	0.046	*Pseudomonadota*	*f Sphingomonadaceae*
5670	0.48	0.84	0.004	NA	377	25	0.17	0.328	*Myxococcota*	s MED-G138 sp019637095
2600	0.42	0.82	0.006	NA	381	22	0.31	0.058	*Verrucomicrobiota*	*s Synoicihabitans sp023386835*
1223	0.44	0.83	0.005	NA	384	20	0.22	0.194	*Pseudomonadota*	*f Burkholderiaceae C*
2047	0.35	0.85	0.006	NA	385	19	0.36	0.026	*Verrucomicrobiota*	*g Cephaloticoccus*
3775	0.46	0.78	0.005	NA	391	20	0.32	0.055	*Chloroflexota*	*g W-Chloroflexi-9*
3145	0.49	0.76	0.004	NA	392	15	0.38	0.022	*Pseudomonadota*	*f Steroidobacteraceae*
2202	0.36	0.84	0.005	NA	408	22	0.34	0.039	*Planctomycetota*	s JADLFV01 sp020849625

Table S17: **Bacterial ASVs indicative of the nine most suppressive composts in the cucumber-*R. solani* system.** For abbreviations see Table S14

ASV	PBC	ISA	MAS	ALX	Rank	n	rho	p	Phylum	Lowest taxonomy
243	0.65	0.92	0.016	NA	28	37	0.56	0.000	*Actinomycetota*	*s Mycobacterium hassiacum*
434	0.56	0.93	0.013	NA	43	33	0.34	0.037	*Pseudomonadota*	*g Rhodomicrobium*
423	0.62	0.90	0.012	NA	56	36	0.40	0.013	*Planctomycetota*	*s UBA2421 sp002343075*
144	0.50	0.92	0.022	NA	58	35	0.24	0.146	*Pseudomonadota*	*f Steroidobacteraceae*
178	0.50	0.93	0.014	NA	64	34	0.51	0.001	*Actinomycetota*	*g Nonomuraea*
13	0.48	0.92	0.089	0.53	68	37	0.45	0.006	*Gemmatimonadota*	*o Longimicrobiales*
2784	0.74	0.94	0.005	NA	73	30	0.57	0.000	*Actinomycetota*	*o Acidimicrobiales*
803	0.49	0.95	0.012	NA	73	30	0.45	0.005	*Chloroflexota*	*f UBA6265*
394	0.47	0.98	0.019	NA	74	34	0.45	0.005	*Pseudomonadota*	*o Rhizobiales*
921	0.51	0.91	0.012	NA	75	31	0.37	0.022	*unclassified*	*unclassified*
1101	0.53	0.92	0.008	NA	76	35	0.33	0.049	*Pseudomonadota*	*f Steroidobacteraceae*
114	0.65	0.84	0.018	NA	77	37	0.42	0.010	*Gemmatimonadota*	*c Gemmatimonadetes*
379	0.51	0.89	0.014	NA	77	35	0.34	0.039	*Pseudomonadota*	f HTCC2089
45	0.43	1.00	0.061	NA	80	36	0.56	0.000	*Gemmatimonadota*	*o Longimicrobiales*
483	0.63	0.87	0.011	NA	80	35	0.50	0.001	*Actinomycetota*	*f Ilumatobacteraceae*
595	0.58	0.86	0.012	NA	83	26	0.37	0.024	*Actinomycetota*	*f Micromonosporaceae*
609	0.51	0.92	0.008	NA	83	32	0.35	0.036	*Actinomycetota*	*g Actinocorallia*
642	0.53	0.88	0.012	NA	84	33	0.42	0.009	*Pseudomonadota*	*s ZC4RG20 sp017577365*
301	0.45	0.92	0.024	NA	85	31	0.41	0.011	*Actinomycetota*	*c Actinomycetia*
10	0.52	0.85	0.054	NA	86	37	0.52	0.001	*Chloroflexota*	*s Sphaerobacter thermophilus*
346	0.41	0.93	0.024	NA	88	34	0.55	0.000	*Pseudomonadota*	*c Gammaproteobacteria*
736	0.49	0.92	0.010	NA	88	34	0.37	0.022	*Pseudomonadota*	*f SG8-39*
4	0.51	0.85	0.065	NA	88	37	0.20	0.242	*Chloroflexota*	*s Aggregatilinea lenta*
1327	0.49	0.90	0.013	NA	89	31	0.37	0.022	*unclassified*	*unclassified*
518	0.52	0.89	0.009	NA	92	35	0.31	0.061	*Deinococcota*	*g JAAYYF01*
8464	0.60	0.91	0.005	NA	93	32	0.59	0.000	*Chloroflexota*	*s Sphaerobacter thermophilus*
298	0.47	0.90	0.019	NA	94	29	0.52	0.001	*Bacteroidota*	*s Anseongella ginsenosidimutans*
3220	0.74	0.89	0.005	NA	95	32	0.58	0.000	*Actinomycetota*	*c Acidimicrobiia*
277	0.51	0.86	0.014	NA	95	35	0.38	0.021	*Acidobacteriota*	*g CADEFD01*
398	0.46	0.91	0.017	NA	96	35	0.53	0.001	*Actinomycetota*	*f ZC4RG35*
891	0.49	0.89	0.012	NA	96	31	0.42	0.009	*Planctomycetota*	*f Lacipirellulaceae*
1249	0.51	0.89	0.008	NA	100	31	0.36	0.027	*Pseudomonadota*	*g Hyphomicrobium C*
1313	0.59	0.85	0.009	NA	102	27	0.42	0.009	*Pseudomonadota*	*g Terrihabitans*
1013	0.49	0.91	0.008	NA	103	34	0.49	0.002	*Pseudomonadota*	*c Alphaproteobacteria*
368	0.48	0.88	0.016	0.56	103	36	0.37	0.025	*Bacillota G*	*g Capillibacterium*
1372	0.52	0.88	0.008	NA	103	28	0.36	0.027	*unclassified*	*unclassified*
788	0.48	0.93	0.008	NA	104	32	0.46	0.005	*Bacteroidota*	*f Cyclobacteriaceae*
102	0.56	0.83	0.012	NA	109	37	0.45	0.005	*Actinomycetota*	*s Thermomonospora curvata*
125	0.49	0.84	0.020	NA	114	37	0.40	0.014	*Actinomycetota*	*s ZC4RG17 sp017577575*
537	0.45	0.92	0.009	NA	116	34	0.28	0.095	*Chloroflexota*	*f Anaerolineaceae*
1416	0.55	0.87	0.006	NA	117	32	0.49	0.002	*Actinomycetota*	*f Miltoncostaeaceae*
658	0.49	0.88	0.008	NA	119	31	0.46	0.004	*Actinomycetota*	*g Nonomuraea*

Continued on next page

ASV	PBC	ISA	MAS	ALX	Rank	n	rho	p	Phylum	Lowest taxonomy
900	0.52	0.79	0.014	NA	123	23	0.31	0.058	*Pseudomonadota*	*g Ga0077530*
105	0.55	0.81	0.010	NA	127	37	0.44	0.007	*Actinomycetota*	*s Thermomonospora curvata*
2729	0.55	0.86	0.005	NA	128	32	0.46	0.004	*Bacillota*	*s Calditerricola satsumensis*
1469	0.49	0.88	0.007	NA	130	33	0.47	0.003	*Planctomycetota*	*s UBA2421 sp002343075*
285	0.53	0.82	0.010	NA	132	36	0.31	0.066	*Chloroflexota*	*p Chloroflexota*
3067	0.64	0.83	0.006	NA	134	34	0.32	0.057	*Chloroflexota*	*s Aggregatilinea lenta*
710	0.54	0.85	0.006	NA	134	35	0.22	0.192	*Pseudomonadota*	*g Filomicrobium*
3809	0.55	0.84	0.006	NA	137	19	0.46	0.005	*Pseudomonadota*	*c Gammaproteobacteria*
3223	0.49	0.87	0.007	NA	137	35	0.31	0.058	*Chloroflexota*	*s Sphaerobacter thermophilus*
794	0.45	0.89	0.008	NA	137	34	0.21	0.221	*Pseudomonadota*	*s Povalibacter uvarum*
1446	0.47	0.91	0.006	NA	138	33	0.51	0.001	*Actinomycetota*	*o Euzebyales*
2055	0.50	0.88	0.005	NA	140	34	0.47	0.003	*Chloroflexota*	*f Thermomicrobiaceae*
791	0.44	0.89	0.008	NA	140	27	0.38	0.020	*Actinomycetota*	*g Streptomyces*
23055	0.58	0.84	0.005	NA	140	31	0.38	0.021	*Chloroflexota*	*s Aggregatilinea lenta*
21324	0.60	0.85	0.003	NA	141	33	0.32	0.053	*Chloroflexota*	*c Anaerolineae*
2403	0.49	0.86	0.007	NA	141	35	0.18	0.284	*Pseudomonadota*	*f Steroidobacteraceae*
269	0.46	0.86	0.011	NA	142	36	0.38	0.022	*Pseudomonadota*	*o Rhizobiales*
749	0.38	0.86	0.015	NA	143	26	0.57	0.000	*Acidobacteriota*	*f SCN-69-37*
2887	0.60	0.83	0.005	NA	144	20	0.59	0.000	*Gemmatimonadota*	*o Longimicrobiales*
1370	0.49	0.87	0.006	NA	144	30	0.49	0.002	*Actinomycetota*	*s VFJN01 sp009694375*
4478	0.46	0.86	0.009	NA	147	29	0.40	0.015	*Gemmatimonadota*	*c Gemmatimonadetes*
2570	0.55	0.85	0.003	NA	149	37	0.47	0.003	*Bacillota A*	*s Caldicoprobacter algeriensis*
2130	0.43	0.90	0.007	NA	149	29	0.41	0.013	*Pseudomonadota*	*c Alphaproteobacteria*
1362	0.54	0.83	0.005	NA	157	34	0.36	0.029	*Actinomycetota*	*o Acidimicrobiales*
21550	0.49	0.84	0.007	NA	157	35	0.24	0.150	*Chloroflexota*	*c Anaerolineae*
523	0.40	0.84	0.013	NA	162	32	0.52	0.001	*Bacteroidota*	*f Cyclobacteriaceae*
3249	0.49	0.85	0.005	NA	163	32	0.31	0.059	*Bacillota A*	*c Clostridia*
3191	0.54	0.77	0.005	NA	167	15	0.49	0.002	*Pseudomonadota*	*s Sinorhizobium fredii*
1024	0.41	0.88	0.006	NA	169	29	0.22	0.188	*Myxococcota*	*f GCA-2862545*
694	0.41	0.74	0.016	NA	170	17	0.53	0.001	*Planctomycetota*	*g Tautonia*
1067	0.48	0.84	0.007	NA	170	26	0.51	0.001	*Actinomycetota*	*g Nonomuraea*
1870	0.46	0.86	0.005	NA	176	25	0.41	0.012	*Pseudomonadota*	*f Sphingomonadaceae*
1594	0.44	0.85	0.007	NA	177	24	0.42	0.010	*Bacteroidota*	*s Flavipsychrobacter sp020636495*
4759	0.50	0.84	0.004	NA	178	35	0.39	0.017	*Actinomycetota*	*s ZC4RG17 sp017577575*
3791	0.51	0.82	0.004	NA	182	21	0.25	0.138	*Bacillota G*	*g Capillibacterium*
2106	0.47	0.73	0.008	NA	183	15	0.36	0.028	*Pseudomonadota*	*s Palsa-892 sp022844205*
23950	0.51	0.82	0.003	NA	184	29	0.33	0.047	*Chloroflexota*	*s Aggregatilinea lenta*
25535	0.40	0.79	0.008	NA	197	23	0.49	0.002	*unclassified*	*unclassified*

References

Alef, K., Nannipieri, P., 1995. 5 - estimation of microbial activities, in: Methods in Applied Soil Microbiology and Biochemistry. Academic Press, London, pp. 193–270. doi:doi:10.1016/B978-012513840-6/50020-3.

Antoniou, A., Tsolakidou, M.D., Stringlis, I.A., Pantelides, I.S., 2017. Rhizosphere microbiome recruited from a suppressive compost improves plant fitness and increases protection against vascular wilt pathogens of tomato. Frontiers in plant science 8, 2022. doi:doi:10.3389/fpls.2017.02022.

Bekkar, A.A., Zaim, S., 2024. Newly isolated brevundimonas naejangsanensis as a biocontrol agent against fusarium redolens the causal of fusarium yellows of chickpea. Folia Microbiologica , 1–12doi:doi:10.1007/s12223-023-01126-z.

Blaya, J., Marhuenda, F.C., Pascual, J.A., Ros, M., 2016. Microbiota characterization of compost using omics approaches opens new perspectives for phytophthora root rot control. PloS one 11, e0158048. doi:doi:10.1371/journal.pone.0158048.

Bohrer, R.E.G., Carissimi, E., Guerra, D., da Silva, D.M., de Souza, E.L., de Vasconcelos, M.C., Wolf, D.B., de Assis Lawisch Rodriguez, A., 2023. Evaluation of bacterial diversity in a swine manure composting system contaminated with veterinary antibiotics (vas). Archives of Microbiology 205, 85. doi:doi:10.1007/s00203-022-03382-y.

Borker, S.S., Thakur, A., Kumar, S., Kumari, S., Kumar, R., Kumar, S., 2021. Comparative genomics and physiological investigation supported safety, cold adaptation, efficient hydrolytic and plant growth-promoting potential of psychrotrophic glutamicibacter arilaitensis ljh19, isolated from night-soil compost. BMC genomics 22, 307. doi:doi:10.1186/s12864-021-07632-z7.

Cáceres, M.D., Legendre, P., 2009. Associations between species and groups of sites: indices and statistical inference. Ecology 90, 3566–3574. doi:doi:10.1890/08-1823.1.

Cai, R., Zuo, S., Cao, X., Xu, C., 2023. Carbon and nitrogen transformation mechanism and functional microorganisms of sheep manure composting on the qinghai-tibet plateau under different moisture content. Journal of Environmental Chemical Engineering 11, 111341. doi:doi:10.1016/j.jece.2023.111341.

Cao, Y., Shen, Z., Zhang, N., Deng, X., Thomashow, L.S., Lidbury, I., Liu, H., Li, R., Shen, Q., Kowalchuk, G.A., 2024. Phosphorus availability influences disease-suppressive soil microbiome through plant-microbe interactions. Microbiome 12, 1–18. doi:doi:10.1186/s40168-024-01906-w.

Carrión, V.J., Perez-Jaramillo, J., Cordovez, V., Tracanna, V., De Hollander, M., Ruiz-Buck, D., Mendes, L.W., van Ijcken, W.F., Gomez-Exposito, R., Elsayed, S.S., et al., 2019. Pathogen-induced activation of disease-suppressive functions in the endophytic root microbiome. Science 366, 606–612.

Charest, M.H., Beauchamp, C.J., Antoun, H., 2005. Effects of the humic substances of de-inking paper sludge on the antagonism between two compost bacteria and pythium ultimum. FEMS Microbiology Ecology 52, 219–227. doi:doi:10.1016/j.femsec.2004.11.017.

Che, J., Bai, Y., Li, X., Ye, J., Liao, H., Cui, P., Yu, Z., Zhou, S., 2021. Linking microbial community structure with molecular composition of dissolved organic matter during an industrial-scale composting. Journal of hazardous materials 405, 124281. doi:doi:10.1016/j.jhazmat.2020.124281.

De Corato, U., Patruno, L., Avella, N., Lacolla, G., Cucci, G., 2019. Composts from green sources show an increased suppressiveness to soilborne plant pathogenic fungi: Relationships between physicochemical properties, disease suppression, and the microbiome. Crop Protection 124, 104870. doi:doi:10.1016/j.cropro.2019.104870.

Deng, X., Zhang, N., Li, Y., Zhu, C., Qu, B., Liu, H., Li, R., Bai, Y., Shen, Q., Falcao Salles, J., 2022. Bio-organic soil amendment promotes the suppression of ralstonia solanacearum by inducing changes in the functionality and composition of rhizosphere bacterial communities. New Phytologist 235, 1558–1574. doi:doi:10.1111/nph.18221.

van Dijk, B., Buffard, P., Farr, A.D., Giersdorf, F., Meijer, J., Dutilh, B.E., Rainey, P.B., 2023. Identifying and tracking mobile elements in evolving compost communities yields insights into the nanobiome. ISME communications 3, 90. doi:doi:10.1038/s43705-023-00294-w.

Ding, J., Wang, N., Liu, P., Liu, B., Zhu, Y., Mao, J., Wang, Y., Ding, X., Yang, H., Wei, Y., et al., 2023. Bacterial wilt suppressive composts: Significance of rhizosphere microbiome. Waste Management 169, 179–185. doi:doi:10.1016/j.wasman.2023.07.011.

Drummond, L., Maher, W., 1995. Determination of phosphorus in aqueous solution via formation of the phosphoantimonylmolybdenum blue complex. re-examination of optimum conditions for the analysis of phosphate. Analytica Chimica Acta 302, 69–74. doi:doi:10.1016/0003-2670(94)00429-P.

Fan, B., Zhao, C., Zhao, L., Wang, M., Sun, N., Li, Z., Yang, F., 2024. Biochar application can enhance phosphorus solubilization by strengthening redox properties of humic reducing microorganisms during composting. Bioresource Technology 395, 130329. doi:doi:10.1016/j.biortech.2024.130329.

Fernandes, A.D., Reid, J.N., Macklaim, J.M., McMurrough, T.A., Edgell, D.R., Gloor, G.B., 2014. Unifying the analysis of high-throughput sequencing datasets: characterizing rna-seq, 16s rrna gene sequencing and selective growth experiments by compositional data analysis. Microbiome 2, 1–13. doi:doi:10.1186/2049-2618-2-15.

Fujiwara, K., Iida, Y., Someya, N., Takano, M., Ohnishi, J., Terami, F., Shinohara, M., 2016. Emergence of antagonism against the pathogenic fungus fusarium oxysporum by interplay among non-antagonistic bacteria in a hydroponics using multiple parallel mineralization. Journal of Phytopathology 164, 853–862. doi:doi:10.1111/jph.12504.

Gal, C., Frenzel, W., Möller, J., 2004. Re-examination of the cadmium reduction method and optimisation of conditions for the determination of nitrate by flow injection analysis. Microchimica Acta 146, 155–164. doi:doi:10.1007/s00604-004-0193-7.

Gao, C.H., Dusa, A., 2024. ggVennDiagram: A 'ggplot2' Implement of Venn Diagram. URL: `https://CRAN.R-project.org/package=ggVennDiagram`. r package version 1.5.2.

Gao, X., Xu, Z., Zhang, L., Li, G., Nghiem, L.D., Luo, W., 2024. Assimilatory sulphate reduction by acidogenesis: The key to prevent h2s formation during food and green waste composting for sustainable urbanization. Chemical Engineering Journal , 156149doi:doi:10.1016/j.cej.2024.156149.

Gavande, P.V., Basak, A., Sen, S., Lepcha, K., Murmu, N., Rai, V., Mazumdar, D., Saha, S.P., Das, V., Ghosh, S., 2021. Functional characterization of thermotolerant microbial consortium for lignocellulolytic enzymes with central role of firmicutes in rice

straw depolymerization. Scientific reports 11, 3032. doi:doi:10.1038/s41598-021-82163-x.

Gu, W., Lu, Y., Tan, Z., Xu, P., Xie, K., Li, X., Sun, L., 2017. Fungi diversity from different depths and times in chicken manure waste static aerobic composting. Bioresource Technology 239, 447–453. doi:doi:10.1016/j.biortech.2017.04.047.

Gu, Y., Banerjee, S., Dini-Andreote, F., Xu, Y., Shen, Q., Jousset, A., Wei, Z., 2022. Small changes in rhizosphere microbiome composition predict disease outcomes earlier than pathogen density variations. The ISME Journal 16, 2448–2456. doi:doi:10.1038/s41396-022-01290-z.

Hernández-Lara, A., Ros, M., Cuartero, J., Bustamante, M.Á., Moral, R., Andreu-Rodríguez, F.J., Fernández, J.A., Egea-Gilabert, C., Pascual, J.A., 2022. Bacterial and fungal community dynamics during different stages of agro-industrial waste composting and its relationship with compost suppressiveness. Science of the Total Environment 805, 150330. doi:doi:10.1016/j.scitotenv.2021.150330.

Hu, L., Xia, M., Gao, X., Huo, Y.X., Yang, Y., 2021a. Cellulosimicrobium composti sp. nov., a thermophilic bacterium isolated from compost. International Journal of Systematic and Evolutionary Microbiology 71, 004905. doi:doi:10.1099/ijsem.0.004905.

Hu, T., Wang, X., Zhen, L., Gu, J., Song, Z., Sun, W., Xie, J., 2021b. Succession of diazotroph community and functional gene response to inoculating swine manure compost with a lignocellulose-degrading consortium. Bioresource Technology 337, 125469. doi:doi:10.1016/j.biortech.2021.125469.

Jaiswal, A.K., Elad, Y., Paudel, I., Graber, E.R., Cytryn, E., Frenkel, O., 2017. Linking the belowground microbial composition, diversity and activity to soilborne disease suppression and growth promotion of tomato amended with biochar. Scientific reports 7, 44382. doi:doi:10.1038/srep44382.

Ji, Y., Cao, Y., Wang, Y., Wang, C., Qin, Z., Cai, W., Yang, Y., Yan, S., Guo, X., 2023. Effects of adding lignocellulose-degrading microbial agents and biochar on nitrogen metabolism and microbial community succession during pig manure composting. Environmental Research 239, 117400. doi:doi:10.1016/j.envres.2023.117400.

Jin, L., Kim, K.K., An, K.G., Oh, H.M., Lee, S.T., 2012. Arenimonas daejeonensis sp. nov., isolated from compost. International journal of systematic and evolutionary microbiology 62, 1674–1678. doi:doi:10.1099/ijs.0.034868-0.

Khatri, S., Chaudhary, P., Shivay, Y.S., Sharma, S., 2023. Role of fungi in imparting general disease suppressiveness in soil from organic field. Microbial Ecology 86, 2047–2059. doi:doi:10.1007/s00248-023-02211-z.

Kim, J.J., Kanaya, E., Weon, H.Y., Koga, Y., Takano, K., Dunfield, P.F., Kwon, S.W., Kanaya, S., 2012. Flavobacterium compostarboris sp. nov., isolated from leaf-and-branch compost, and emended descriptions of flavobacterium hercynium, flavobacterium resistens and flavobacterium johnsoniae. International journal of systematic and evolutionary microbiology 62, 2018–2024. doi:doi:10.1099/ijs.0.032920-0.

Kim, K.H., Ten, L.N., Liu, Q.M., Im, W.T., Lee, S.T., 2006. Sphingobacterium daejeonense sp. nov., isolated from a compost sample. International journal of systematic and evolutionary microbiology 56, 2031–2036. doi:doi:10.1099/ijs.0.64406-0.

Kim, S.J., Weon, H.Y., Kim, Y.S., Yoo, S.H., Kim, B.Y., Anandham, R., Kwon, S.W., 2010. Parapedobacter luteus sp. nov. and parapedobacter composti sp. nov., isolated from cotton waste compost. International journal of systematic and evolutionary microbiology 60, 1849–1853. doi:doi:10.1099/ijs.0.013318-0.

Kinkel, L.L., Schlatter, D.C., Bakker, M.G., Arenz, B.E., 2012. Streptomyces competition and co-evolution in relation to plant disease suppression. Research in microbiology 163, 490–499. doi:doi:10.1016/j.resmic.2012.07.005.

Krom, M.D., 1980. Spectrophotometric determination of ammonia: a study of a modified berthelot reaction using salicylate and dichloroisocyanurate. Analyst 105, 305–316. doi:doi:10.1039/AN9800500305.

Kudjordjie, E.N., Sapkota, R., Nicolaisen, M., 2023. Host-and fusarium-adapted bacterial consortia alter microbial community structures in arabidopsis roots and suppress fusarium oxysporum. Phytobiomes Journal 8, 85–96. doi:doi:10.1094/PBIOMES-09-22-0062-MF.

Kurakov, A., Bilanenko, E., 2023. Dynamics of mycobiota during composting of cow manure and straw. Eurasian Soil Science 56, 453–469. doi:doi:10.1134/S1064229322602554.

Kurm, V., Visser, J., Schilder, M., Nijhuis, E., Postma, J., Korthals, G., 2023. Soil suppressiveness against pythium ultimum and rhizoctonia solani in two land management systems and eleven soil health treatments. Microbial Ecology 86, 1709–1724. doi:doi:10.1007/s00248-023-02215-9.

Li, H., Yang, Z., Zhang, C., Shang, W., Zhang, T., Chang, X., Wu, Z., He, Y., 2024. Effect of microbial inoculum on composting efficiency in the composting process of spent mushroom substrate and chicken manure. Journal of Environmental Management 353, 120145. doi:doi:10.1016/j.jenvman.2024.120145.

Li, J., Chen, Y.T., Xia, Z.Y., Gou, M., Sun, Z.Y., Tang, Y.Q., 2020. Changes in bacterial communities during a pilot-scale composting process of dairy manure. Journal of Environmental Engineering 146, 04020095. doi:doi:10.1061/(ASCE)EE.1943-7870.0001774.

Li, Z., Bai, X., Jiao, S., Li, Y., Li, P., Yang, Y., Zhang, H., Wei, G., 2021. A simplified synthetic community rescues astragalus mongholicus from root rot disease by activating plant-induced systemic resistance. Microbiome 9, 1–20. doi:doi:10.1186/s40168-021-01169-9.

Lima-Junior, J.D., Viana-Niero, C., Conde Oliveira, D.V., Machado, G.E., Rabello, M.C.d.S., Martins-Junior, J., Martins, L.F., Digiampietri, L.A., Da Silva, A.M., Setubal, J.C., et al., 2016. Characterization of mycobacteria and mycobacteriophages isolated from compost at the são paulo zoo park foundation in brazil and creation of the new mycobacteriophage cluster u. BMC microbiology 16, 1–15. doi:doi:10.1186/s12866-016-0734-3.

Lin, J., Yang, G., Tang, J., Li, Z., Yu, Z., Zhuang, L., 2019. Pseudoxanthomonas composti sp. nov., isolated from compost. Antonie Van Leeuwenhoek 112, 1213–1219. doi:doi:10.1007/s10482-019-01253-z.

Liu, Y., Zhang, H., Wang, J., Gao, W., Sun, X., Xiong, Q., Shu, X., Miao, Y., Shen, Q., Xun, W., et al., 2024. Nonpathogenic pseudomonas syringae derivatives and

its metabolites trigger the plant "cry for help" response to assemble disease suppressing and growth promoting rhizomicrobiome. Nature Communications 15, 1907. doi:doi:10.1038/s41467-024-46254-3.

Lu, X., Yang, Y., Hong, C., Zhu, W., Yao, Y., Zhu, F., Hong, L., Wang, W., 2022. Optimization of vegetable waste composting and the exploration of microbial mechanisms related to fungal communities during composting. Journal of Environmental Management 319, 115694. doi:doi:10.1016/j.jenvman.2022.115694.

Lv, N., Tao, C., Ou, Y., Wang, J., Deng, X., Liu, H., Shen, Z., Li, R., Shen, Q., 2023. Root-associated antagonistic pseudomonas spp. contribute to soil suppressiveness against banana fusarium wilt disease of banana. Microbiology Spectrum 11, e03525–22. doi:doi:10.1128/spectrum.03525-22.

Mallick, H., Rahnavard, A., McIver, L.J., Ma, S., Zhang, Y., Nguyen, L.H., Tickle, T.L., Weingart, G., Ren, B., Schwager, E.H., et al., 2021. Multivariable association discovery in population-scale meta-omics studies. PLoS computational biology 17, e1009442. doi:doi:10.1371/journal.pcbi.1009442.

Matsuda, Y., Iida, Y., Shinogi, T., Kakutani, K., Nonomura, T., Toyoda, H., 2001. In vitro suppression of mycelial growth of fusarium oxysporum by extracellular chitosanase of sphingobacterium multivorum and cloning of the chitosanase gene csn sm1. Journal of general plant pathology 67, 318–324. doi:doi:10.1007/PL00013039.

Mayerhofer, J., Thuerig, B., Oberhaensli, T., Enderle, E., Lutz, S., Ahrens, C.H., Fuchs, J.G., Widmer, F., 2021. Indicative bacterial communities and taxa of disease-suppressing and growth-promoting composts and their associations to the rhizoplane. FEMS Microbiology Ecology 97, fiab134. doi:doi:10.1093/femsec/fiab134.

Meena, M., Swapnil, P., Zehra, A., Aamir, M., Dubey, M.K., Goutam, J., Upadhyay, R., 2017. Beneficial microbes for disease suppression and plant growth promotion. Plant-Microbe Interactions in Agro-Ecological Perspectives: Volume 2: Microbial Interactions and Agro-Ecological Impacts , 395–432doi:doi:10.1007/978-981-10-6593-4_16.

Messiha, N.A., Elhalag, K.M., Abd El-Rahman, A.F., Abdelaziz, A.M., ElBadry, N., Hussien, A., 2023. Enhancement of soil suppressive potential to bacterial wilt disease

caused by ralstonia solanacearum. Archives of Phytopathology and Plant Protection 56, 1127–1165. doi:doi:10.1080/03235408.2023.2267668.

Moriya, T., Hikota, T., Yumoto, I., Ito, T., Terui, Y., Yamagishi, A., Oshima, T., 2011. Calditerricola satsumensis gen. nov., sp. nov. and calditerricola yamamurae sp. nov., extreme thermophiles isolated from a high-temperature compost. International Journal of Systematic and EvolutionaryMicrobiology 61, 631–636. doi:doi:10.1099/ijs.0.018416-0.

Nearing, J.T., Douglas, G.M., Hayes, M.G., MacDonald, J., Desai, D.K., Allward, N., Jones, C.M., Wright, R.J., Dhanani, A.S., Comeau, A.M., et al., 2022. Microbiome differential abundance methods produce different results across 38 datasets. Nature communications 13, 342. doi:doi:10.1038/s41467-022-28034-z.

Oka, Y., 2010. Mechanisms of nematode suppression by organic soil amendments—a review. Applied Soil Ecology 44, 101–115. doi:doi:10.1016/j.apsoil.2009.11.003.

Oshins, C., Michel, F., Louis, P., Richard, T.L., Rynk, R., 2022. The composting process, in: The composting handbook. Elsevier, pp. 51–101. doi:doi:10.1016/B978-0-323-85602-7.00008-X.

Ou, Y., Penton, C.R., Geisen, S., Shen, Z., Sun, Y., Lv, N., Wang, B., Ruan, Y., Xiong, W., Li, R., et al., 2019. Deciphering underlying drivers of disease suppressiveness against pathogenic fusarium oxysporum. Frontiers in microbiology 10, 2535. doi:doi:10.3389/fmicb.2019.02535.

Parab, C., Yadav, K.D., Prajapati, V., 2023. Genomics and microbial dynamics in green waste composting: A mini review. Ecological Genetics and Genomics 29, 100206. doi:doi:10.1016/j.egg.2023.100206.

Postma, J., Geraats, B.P., Pastoor, R., Van Elsas, J.D., 2005. Characterization of the microbial community involved in the suppression of pythium aphanidermatum in cucumber grown on rockwool. Phytopathology 95, 808–818. doi:doi:10.1094/PHYTO-95-0808.

Ren, L., Huang, X., Min, H., Wang, H., Xie, Y., Zou, H., Qiao, C., Wu, W., 2023. Different ratios of raw material triggered composting maturity associated with bacterial

community co-occurrence patterns. Environmental Science and Pollution Research 30, 62532–62543. doi:doi:10.1007/s11356-023-26468-6.

Ryder, M., Jones, D., et al., 1991. Biological control of crown gall using agrobacterium strains k84 and k1026. Australian Journal of Plant Physiology (Australia) 18.

Salamoni, S.P., Mann, M.B., Campos, F.S., Franco, A.C., Germani, J.C., Van Der Sand, S.T., 2010. Preliminary characterization of some streptomyces species isolated from a composting process and their antimicrobial potential. World Journal of Microbiology and Biotechnology 26, 1847–1856. doi:doi:10.1007/s11274-010-0366-y.

Scotti, R., Mitchell, A.L., Pane, C., Finn, R.D., Zaccardelli, M., 2020. Microbiota characterization of agricultural green waste-based suppressive composts using omics and classic approaches. Agriculture 10, 61. doi:doi:10.3390/agriculture10030061.

Shen, T., Lei, Y., Pu, X., Zhang, S., Du, Y., 2021. Identification and application of streptomyces microflavus g33 in compost to suppress tomato bacterial wilt disease. Applied Soil Ecology 157, 103724. doi:doi:10.1016/j.apsoil.2020.103724.

Shen, Z., Xue, C., Penton, C.R., Thomashow, L.S., Zhang, N., Wang, B., Ruan, Y., Li, R., Shen, Q., 2019. Suppression of banana panama disease induced by soil microbiome reconstruction through an integrated agricultural strategy. Soil Biology and Biochemistry 128, 164–174. doi:doi:10.1016/j.soilbio.2018.10.016.

Siegel-Hertz, K., Edel-Hermann, V., Chapelle, E., Terrat, S., Raaijmakers, J.M., Steinberg, C., 2018. Comparative microbiome analysis of a fusarium wilt suppressive soil and a fusarium wilt conducive soil from the châteaurenard region. Frontiers in Microbiology 9, 568. doi:doi:10.3389/fmicb.2018.00568.

Snelders, N.C., Rovenich, H., Petti, G.C., Rocafort, M., van den Berg, G.C., Vorholt, J.A., Mesters, J.R., Seidl, M.F., Nijland, R., Thomma, B.P., 2020. Microbiome manipulation by a soil-borne fungal plant pathogen using effector proteins. Nature Plants 6, 1365–1374. doi:doi:10.1038/s41477-020-00799-5.

Storey, S., Chualain, D.N., Doyle, O., Clipson, N., Doyle, E., 2015. Comparison of bacterial succession in green waste composts amended with inorganic fer-

tiliser and wastewater treatment plant sludge. Bioresource technology 179, 71–77. doi:doi:10.1016/j.biortech.2014.11.107.

Sun, Y., Men, M., Xu, B., Meng, Q., Bello, A., Xu, X., Huang, X., 2019. Assessing key microbial communities determining nitrogen transformation in composting of cow manure using illumina high-throughput sequencing. Waste Management 92, 59–67. doi:doi:10.1016/j.wasman.2019.05.007.

Tian, W., Sun, Q., Xu, D., Zhang, Z., Chen, D., Li, C., Shen, Q., Shen, B., 2013. Succession of bacterial communities during composting process as detected by 16s rrna clone libraries analysis. International Biodeterioration & Biodegradation 78, 58–66. doi:doi:10.1016/j.ibiod.2012.12.008.

Ting, A.S.Y., Hoon, T.S., Kay, W.M., Ern, C.L., 2010. Characterization of actinobacteria with antifungal potential against fusarium crown-rot pathogen. Pest Technology 4, 65–69.

Tondello, A., Fasolo, A., Marcato, S., Treu, L., Bonato, T., Zanardi, W., Concheri, G., Squartini, A., Baldan, B., 2022. Characterization of bacterial communities isolated from municipal waste compost and screening of their plant-interactive phenotypes. Science of The Total Environment 806, 150592. doi:doi:10.1016/j.scitotenv.2021.150592.

Tortosa, G., Fernández-González, A.J., Lasa, A.V., Aranda, E., Torralbo, F., González-Murua, C., Fernández-López, M., Benítez, E., Bedmar, E.J., 2021. Involvement of the metabolically active bacteria in the organic matter degradation during olive mill waste composting. Science of The Total Environment 789, 147975. doi:doi:10.1016/j.scitotenv.2021.147975.

Vaz-Moreira, I., Faria, C., Lopes, A.R., Svensson, L., Falsen, E., Moore, E.R., Ferreira, A.C.S., Nunes, O.C., Manaia, C.M., 2009. Sphingobium vermicomposti sp. nov., isolated from vermicompost. International journal of systematic and evolutionary microbiology 59, 3145–3149. doi:doi:10.1099/ijs.0.006163-0.

Vaz-Moreira, I., Faria, C., Lopes, A.R., Svensson, L.A., Moore, E.R., Nunes, O.C., Manaia, C.M., 2010. Shinella fusca sp. nov., isolated from domestic waste compost. International journal of systematic and evolutionary microbiology 60, 144–148. doi:doi:10.1099/ijs.0.009498-0.

Wang, H., Han, L., Feng, J., Zhang, X., 2015. Evaluation of two streptomyces spp. and compost for growth promotion and biocontrol potential against rhizoctonia solani on pepper. Biocontrol Science and Technology 25, 852–857. doi:doi:10.1080/09583157.2015.1015485.

Wang, S.P., Sun, Z.Y., Wang, S.T., Yuan, H.W., An, M.Z., Xia, Z.Y., Tang, Y.Q., Shen, C.H., Kida, K., 2022a. Bacterial community structure and metabolic function succession during the composting of distilled grain waste. Applied Biochemistry and Biotechnology , 1–17doi:doi:10.1007/s12010-021-03731-5.

Wang, Z., Zhao, M., Xie, J., Wang, Z., Tsui, T.H., Ren, X., Zhang, Z., Wang, Q., 2022b. Insight into the fraction variations of selenium and their effects on humification during composting. Bioresource Technology 364, 128050.

Wei, W., Deng, H., Li, G., Wang, X., Lu, J., 2016. Performance study of compounded biocover material for methane removal based on cattle manure compost. Environmental Technology 37, 535–545. doi:doi:10.1080/09593330.2015.1074722.

Wen, T., Ding, Z., Thomashow, L.S., Hale, L., Yang, S., Xie, P., Liu, X., Wang, H., Shen, Q., Yuan, J., 2023. Deciphering the mechanism of fungal pathogen-induced disease-suppressive soil. New Phytologist 238, 2634–2650. doi:doi:10.1111/nph.18886.

Weon, H.Y., Kim, B.Y., Kwon, S.W., Go, S.J., Koo, B.S., Stackebrandt, E., 2008. Phenylobacterium composti sp. nov., isolated from cotton waste compost in korea. International journal of systematic and evolutionary microbiology 58, 2301–2304. doi:doi:10.1099/ijs.0.65530-0.

Wu, H., Liu, B., 2016. Nonomuraea thermotolerans sp. nov., a thermotolerant actinomycete isolated from mushroom compost. International Journal of Systematic and Evolutionary Microbiology 66, 894–900. doi:doi:10.1099/ijsem.0.000809.

Wu, H., Wei, J., Liu, B., 2019. Thermomonospora catenispora sp. nov., isolated from mushroom compost. International Journal of Systematic and Evolutionary Microbiology 69, 2465–2470. doi:doi:10.1099/ijsem.0.003515.

Xiong, W., Li, R., Ren, Y., Liu, C., Zhao, Q., Wu, H., Jousset, A., Shen, Q., 2017. Distinct roles for soil fungal and bacterial communities associated with the suppres-

sion of vanilla fusarium wilt disease. Soil Biology and Biochemistry 107, 198–207. doi:doi:10.1016/j.soilbio.2017.01.010.

Xu, L., Zhang, H., Xing, Y.T., Li, N., Wang, S., Sun, J.Q., 2020. Complete genome sequence of sphingobacterium psychroaquaticum strain sj-25, an aerobic bacterium capable of suppressing fungal pathogens. Current Microbiology 77, 115–122.

Xue, C., Ryan Penton, C., Shen, Z., Zhang, R., Huang, Q., Li, R., Ruan, Y., Shen, Q., 2015. Manipulating the banana rhizosphere microbiome for biological control of panama disease. Scientific reports 5, 11124. doi:doi:10.1038/srep11124.

Yang, H., Lee, J., Cho, K.S., 2023. Dynamics of functional genes and bacterial community during bioremediation of diesel-contaminated soil amended with compost. Journal of Microbiology and Biotechnology 33, 471. doi:doi:10.4014/jmb.2210.10038.

Young, C.C., Kämpfer, P., Chen, W.M., Yen, W.S., Arun, A., Lai, W.A., Shen, F.T., Rekha, P., Lin, K.Y., Chou, J.H., 2007. Luteimonas composti sp. nov., a moderately thermophilic bacterium isolated from food waste. International journal of systematic and evolutionary microbiology 57, 741–744. doi:doi:10.1099/ijs.0.64701-0.

Zainudin, M.H., Mustapha, N.A., Maeda, T., Ramli, N., Sakai, K., Hassan, M., 2020. Biochar enhanced the nitrifying and denitrifying bacterial communities during the composting of poultry manure and rice straw. Waste Management 106, 240–249. doi:doi:10.1016/j.wasman.2020.03.029.

Zhang, X., Zhu, Y., Li, J., Zhu, P., Liang, B., 2021. Exploring dynamics and associations of dominant lignocellulose degraders in tomato stalk composting. Journal of Environmental Management 294, 113162. doi:doi:10.1016/j.jenvman.2021.113162.

Zhao, B., Cao, X., Cai, Z., Zhang, L., Li, D., Zhang, H., Li, S., Sun, X., 2023. Improving suppressive activity of compost on phytopathogenic microbes by inoculation of antagonistic microorganisms for secondary fermentation. Bioresource Technology 367, 128288. doi:doi:10.1016/j.biortech.2022.128288.

Zheng, X., Wang, J., Chen, M., Chen, Y., Chen, Z., Wang, M., Liu, B., 2024. Testing a biocontrol agent consortium for suppression of tomato bacterial wilt

through rhizosphere microecological regulation. Applied Soil Ecology 193, 105155. doi:doi:10.1016/j.apsoil.2023.105155.

Zhong, X.Z., Li, X.X., Zeng, Y., Wang, S.P., Sun, Z.Y., Tang, Y.Q., 2020. Dynamic change of bacterial community during dairy manure composting process revealed by high-throughput sequencing and advanced bioinformatics tools. Bioresource technology 306, 123091. doi:doi:10.1016/j.biortech.2020.123091.
